# Modulation of metastable ensemble dynamics explains optimal coding at moderate arousal in auditory cortex

**Published:** 2024-04-08

**Authors:** Lia Papadopoulos, Suhyun Jo, Kevin Zumwalt, Michael Wehr, David A. McCormick, Luca Mazzucato

**Affiliations:** Institute of Neuroscience, University of Oregon, Eugene, Oregon; Institute of Neuroscience, University of Oregon, Eugene, Oregon; Institute of Neuroscience, University of Oregon, Eugene, Oregon; Institute of Neuroscience, University of Oregon, Eugene, Oregon; Department of Psychology, University of Oregon, Eugene, Oregon; Institute of Neuroscience, University of Oregon, Eugene, Oregon; Department of Biology, University of Oregon, Eugene, Oregon; Institute of Neuroscience, University of Oregon, Eugene, Oregon; Department of Biology, University of Oregon, Eugene, Oregon; Department of Mathematics, University of Oregon, Eugene, Oregon; Department of Physics, University of Oregon, Eugene, Oregon

## Abstract

Performance during perceptual decision-making exhibits an inverted-U relationship with arousal, but the underlying network mechanisms remain unclear. Here, we recorded from auditory cortex (A1) of behaving mice during passive tone presentation, while tracking arousal via pupillometry. We found that tone discriminability in A1 ensembles was optimal at intermediate arousal, revealing a population-level neural correlate of the inverted-U relationship. We explained this arousal-dependent coding using a spiking network model with a clustered architecture. Specifically, we show that optimal stimulus discriminability is achieved near a transition between a multi-attractor phase with metastable cluster dynamics (low arousal) and a single-attractor phase (high arousal). Additional signatures of this transition include arousal-induced reductions of overall neural variability and the extent of stimulus-induced variability quenching, which we observed in the empirical data. Altogether, this study elucidates computational principles underlying interactions between pupil-linked arousal, sensory processing, and neural variability, and suggests a role for phase transitions in explaining nonlinear modulations of cortical computations.

## I. INTRODUCTION

Cognitive function is impacted by fluctuations in brain and behavioral states [1–7]. For example, variations in arousal – generally defined as an animal’s overall level of alertness – play a critical role in the regulation of sensory processing during wakefulness [1–4, 6, 7]. The impacts of arousal are mediated by broadly-projecting neuromodulatory pathways, including the cholinergic and noradrenergic systems [8–12], as well as by thalamocortical pathways [2, 13, 14]. Changes in arousal can also be non-invasively monitored with pupillometry [15–17], and fluctuations in pupil-linked arousal are accompanied by changes in behavioral task performance across multiple sensory modalities and species [1, 18–26].

The relationship between arousal and performance is often discussed in the context of the Yerkes-Dodson “inverted-U” law [27]. This model posits that animals’ performance on difficult tasks should be poor at both low arousal (when inattentive) and high arousal (when anxious), with optimal performance achieved during states of intermediate arousal. The inverted-U law has been particularly well-studied in the context of auditory processing, with examples reported in mice performing sound detection [20] and discrimination tasks [22], and in humans performing auditory oddball [19] and pitch discrimination [18] tasks.

Past work has begun to uncover neural signatures of the inverted-U relationship during auditory processing. In mice trained on a tone-in-noise detection task, evoked responses from auditory cortex were found to be largest and most reliable at intermediate levels of arousal [20]. Broadly consistent with those findings is the observed suppression of sound-evoked responses in auditory cortex during high-arousal states associated with locomotion [28–31]. However, the network-level dynamical principles underlying optimal performance states, especially in regard to population coding of auditory stimuli, remain unclear. To gain mechanistic insight, here we utilize a combination of electrophysiological experiments, network simulations, and theoretical analysis.

Given that neural correlates of the inverted-U relationship have been observed in auditory cortex even without task engagement [20], we examined how arousal impacts neural discriminability of pure tones during passive presentation. To achieve this, we used Neuropixels probes to record activity from ensembles of primary auditory cortex (A1) neurons in awake mice, and simultaneously monitored arousal state with pupillometry. We found that tone frequency was best decoded from A1 ensemble activity during periods of intermediate pupil dilation, in line with an inverted-U relationship. This finding extends previous results on optimal sound detection in auditory cortex [1] to population coding.

To illuminate potential network mechanisms underlying the inverted-U relationship between arousal and neural discriminability, we modeled A1 as a network of spiking neurons arranged in a clustered architecture. As shown previously, this model generates metastable dynamics characterized by the transient activation of neural assemblies [32–34]. By modeling arousal as a modulation of background inputs to the A1 circuit, we show that stimulus decoding accuracy can be controlled by regulating the spontaneous metastable cluster dynamics. Namely, we demonstrate that the inverted-U relationship emerges via a transition from a multi-attractor phase (low arousal condition) to a single-attractor phase (high arousal condition), with optimal stimulus encoding achieved near the transition region. This nonlinear effect was absent in networks with uniform connectivity, and thus relies specifically on the presence of metastable dynamics in the clustered network. The clustered model additionally predicts that spontaneous and evoked neural variability should be reduced at high arousal, as should the amount of stimulus-induced quenching of variability [35]. We found evidence for these predictions in the experimental data, lending support to the proposed network mechanism. As a whole, our results suggest that arousal-induced transitions in the dynamical regime of a cortical circuit may explain key aspects of arousal-dependent stimulus processing and neural variability in auditory cortex.

## II. RESULTS

**FIG. 1.**
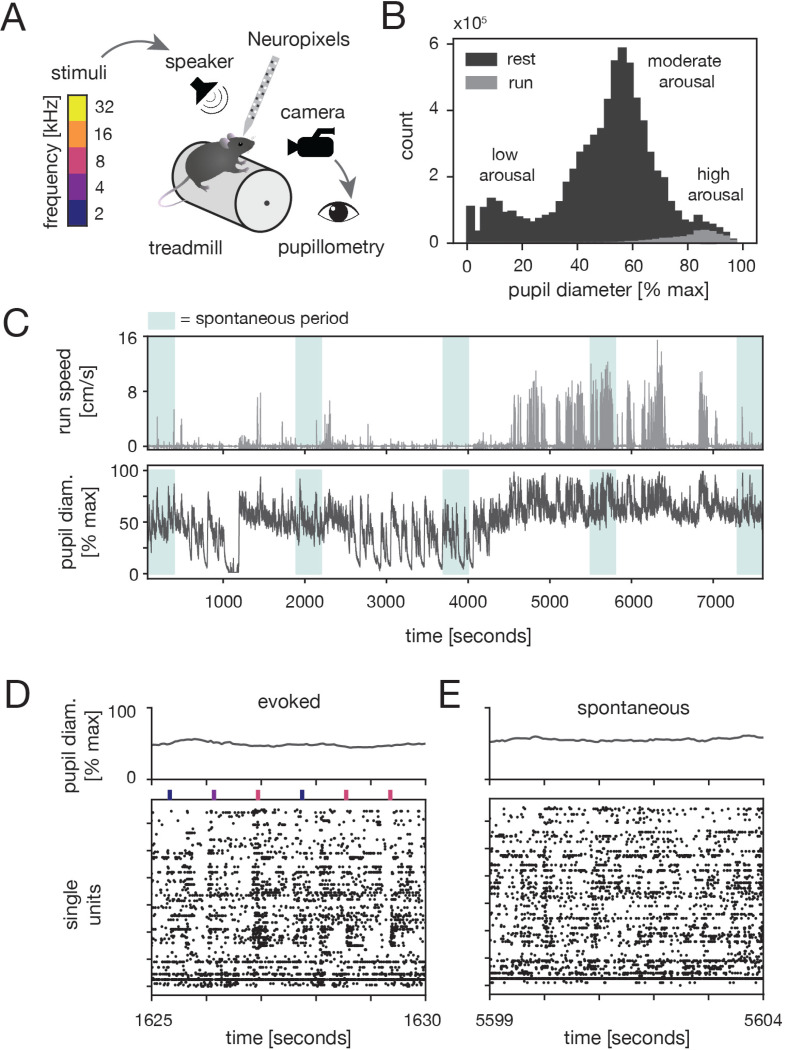
Neuropixels recordings from A1 of awake mice during a range of arousal states. **(A)** Awake, head-fixed mice were situated on a treadmill while neural activity was recorded using a Neuropixels probe. Throughout a session, mice were presented with pure tones of five different frequencies and arousal state was monitored with pupillometry. **(B)** Pupil diameter distributions from an example recording during rest periods (dark gray) or running periods (light gray). **(C)** Running speed and pupil diameter traces from an example recording session. Light green areas indicate spontaneous periods (no stimulus presentation) and white areas indicate evoked periods. **(D)** Pupil diameter trace and population raster across 5 seconds of evoked activity; vertical lines above the raster indicate stimulus onset times and colors correspond to the frequencies in **A**. **(E)** Pupil diameter trace and population raster across 5 seconds of spontaneous activity.

We measured neural activity from A1 of awake, head-fixed mice while simultaneously monitoring locomotion speed and pupil-indexed arousal (Fig. 1A-C; Sec. IV A). Single-unit activity was recorded using Neuropixels probes both during sound presentation (Fig. 1D, “evoked” periods) and in the absence of auditory stimuli (Fig. 1E, “spontaneous” periods). During, evoked periods, mice were presented with 25 ms tones (2, 4, 8, 16, or 32 kHz). A full spectrum of arousal states was thoroughly-sampled in many recordings, and either the lower or upper half of the pupil range was well-sampled in the remaining sessions (Fig. S1).

### A. Encoding of tone frequency in A1 populations is optimal at intermediate arousal

**FIG. 2.**
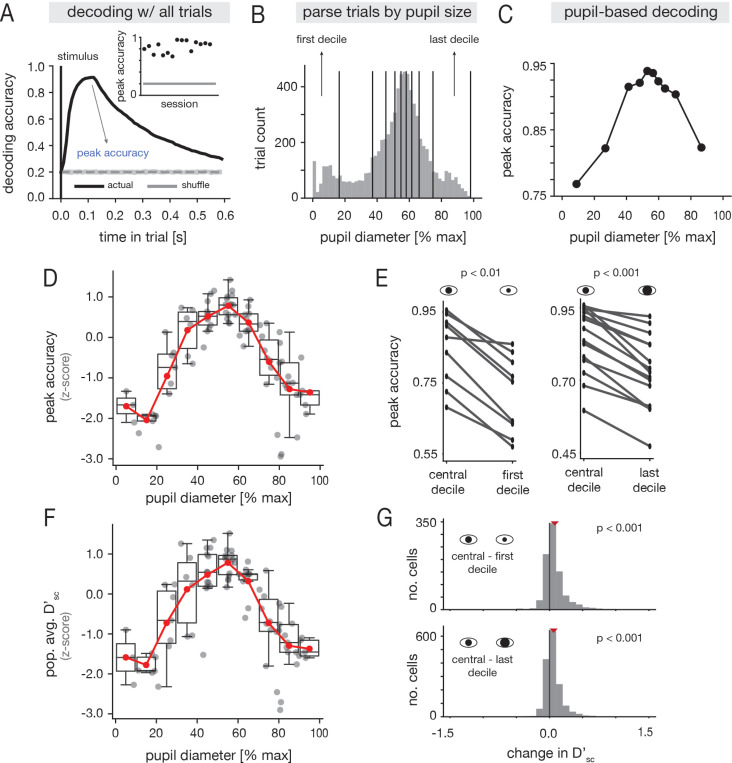
Encoding of tone frequency in A1 populations is enhanced at intermediate arousal. **(A)** Decoding accuracy *vs.* time relative to stimulus onset using all trials from an example session. The light gray area denotes the 5^th^ to 95^th^ percentile range of the shuffled accuracy distribution (Sec. IV C). **Inset:** Peak accuracy in each session. The gray line indicates chance performance. **(B)** Histogram of the pre-stimulus pupil diameter in an example session; black lines indicate deciles. **(C)** Peak accuracy in each pupil decile from **(B)**. **(D)** Peak accuracy (z-scored) *vs.* pupil diameter. Within each session, peak accuracy values were z-scored across pupil deciles. The normalized data was then pooled across sessions (*n* = 15), and binned by pupil diameter. For each bin, we show individual data points (gray), the mean (red), and corresponding boxplot (Sec. IV C 5). **(E) Left:** Peak accuracy in the most central and first pupil decile of a session (*p* < 0.01, *n* = 9 sessions; Wilcoxon signed-rank test). **Right:** Peak accuracy in the most central and last pupil decile of a session (*p* < 0.001, *n* = 15 sessions; Wilcoxon signed-rank test). Only sessions where the first (last) decile was centered at < 25% (> 75%) of maximum dilation were included in the top (bottom) analyses (Sec. IV C 6). **(F)** Same as **(D)** but for the population-averaged $D_{\text{sc}}^{\prime }$ (Sec. IV G). **(G) Top:** Distribution of the difference in $D_{\text{sc}}^{\prime }$ between the most central and first pupil deciles; $D_{\text{sc}}^{\prime }$ was significantly larger for central deciles (Wilcoxon signed-rank test, *p* < 0.001, *n* = 898). **Bottom:** Distribution of the difference in $D_{\text{sc}}^{\prime }$ between the most central and last pupil deciles; $D_{\text{sc}}^{\prime }$ was significantly larger for central deciles (Wilcoxon signed-rank test, *p* < 0.001, *n* = 1555). For the top (bottom) histogram, cells were pooled across all sessions for which the first (last) decile was centered at < 25% (> 75%) of maximum dilation.

To determine if tone frequency was robustly encoded in recorded A1 ensembles, we trained a linear decoder to discriminate between the five tones given single-trial population activity (Sec. IV C). As expected, frequency information could be reliably decoded in all sessions (Fig. 2A; Fig. S3). We next tested whether arousal modulates the encoding of tones in A1. To this end, we grouped trials by pupil diameter (Fig. 2B; Fig. S2 for all sessions), and computed the maximum decoding accuracy in each pupil-based partition (Fig. 2C; Sec. IV C). On average across sessions, decoding performance followed an inverted-U relationship with pupil diameter (Fig. 2D; Fig. S4 for individual sessions), and there was a statistically significant increase in accuracy at mid-range pupil diameters relative to either the lowest or highest diameters ( Fig. 2E; Sec. IV C 6). Moreover, in all sessions, the best performance was achieved at moderate pupil diameters, and the worst performance at low or high pupil diameters (Fig. S5A). The session-averaged decoding performance still exhibited an inverted-U relationship with pupil diameter after excluding locomotion trials (Fig. S6A), though the trend was less pronounced. However, this difference may in part be due the fact that average pupil diameters were smaller without movement data (Fig. S6B). As a whole, these findings indicate that frequency information is best represented in A1 population activity at moderate arousal.

To further understand the population decoding results, we also examined how a single-cell discriminability index $\left(D_{\text{sc}}^{\prime }\right)$ varied with pupil diameter (Sec. IV G). On average across cells and sessions, $D_{\text{sc}}^{\prime }$ followed an inverted-U relationship with pupil diameter, similar to the decoding performance (Fig. 2F; Fig. S7 for individual sessions). At the level of individual units, intermediate pupil diameters were associated with significant increases in $D_{\text{sc}}^{\prime }$ relative to either small or large diameters (Fig. 2G), and at the cell-averaged level in individual recordings, $D_{\text{sc}}^{\prime }$ was always highest at moderate pupil diameter (Fig. S5B). Taken together, these findings suggest that arousal-related modulations of decoding performance at the population-level are accompanied by overall changes in discriminability at the single-neuron level.

### B. Diverse impacts of arousal on spontaneous firing rates can be explained by a network model with heterogeneous modulation of background inputs

**FIG. 3.**
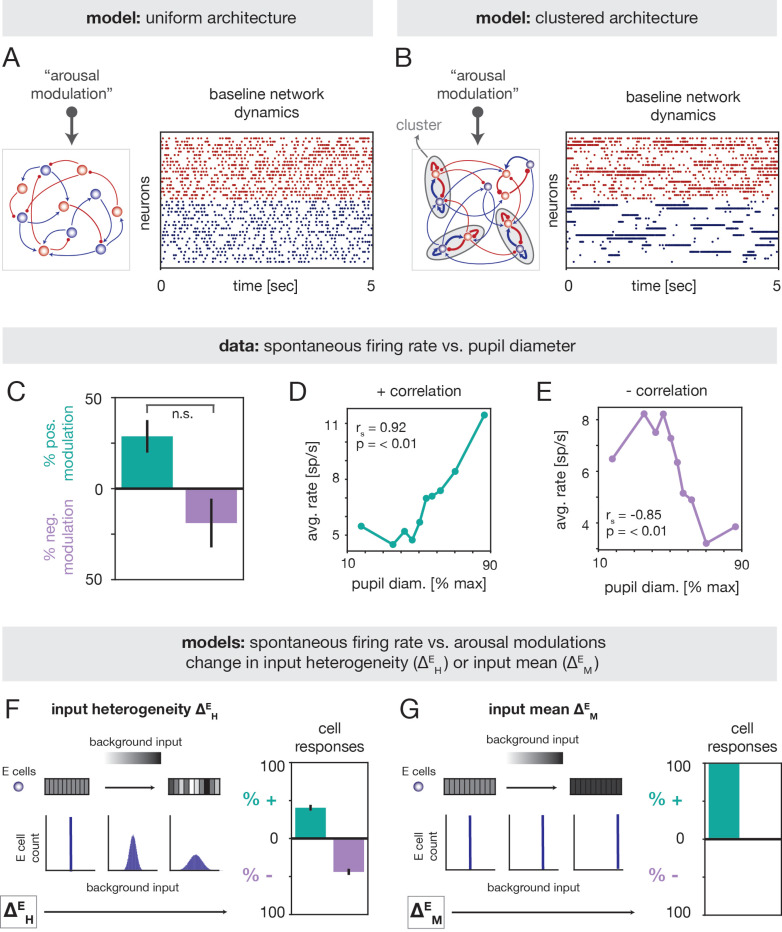
Alternative network models for explaining arousal-dependent modulations of A1 activity. **(A, B)** A1 is modeled as a recurrent network of spiking neurons arranged in either a uniform architecture (**A Left**) or a clustered architecture (**B Left**). In both cases, a change in arousal is implemented as a modulation of background external input to the circuit. Raster plots show baseline network activity for a subset of neurons from either the uniform network (**A Right**) or clustered network (**B Right**). See Sec. IV B for model details. **(C)** Fraction of units whose spontaneous firing rate increases or decreases with pupil diameter in the experimental data; bar heights and error bars indicate the mean ± 1 S.D. across sessions (Sec. IV E). There was no significant difference between the fraction of positively and negatively modulated units (Wilcoxon-signed rank test, *p* = 0.135, *n* = 15). **(D)** A unit whose spontaneous firing rate increases with pupil diameter (Spearman correlation *r*_*s*_ = 0.9, *p* < 0.01). **(E)** A unit whose spontaneous firing rate decreases with pupil diameter (Spearman correlation *r*_*s*_ = −0.05, *p* < 0.01 **(F, G)** Alternative choices for the arousal modulation in the circuit models (Sec. IV B 4). **(F Left)** An increase in arousal is modeled as an increase in the heterogeneity of background inputs across E cells (parameterized by ${\text{Δ}}_{H}^{E}$), while keeping the mean input across cells fixed. Formally, this was achieved by drawing the input to a given E cell from a Gaussian with a fixed mean but increasing variance. **(F Right)** Fraction of all neurons whose spontaneous firing rate increases or decreases with ${\text{Δ}}_{H}^{E}$ in the clustered network (see Fig. S14A for similar results in the uniform network). **(G Left)** An increase in arousal is modeled as a uniform increase in the strength of the background input to E cells (parameterized by ${\text{Δ}}_{M}^{E}$). **(G Right)** Fraction of all neurons whose spontaneous firing rate increases or decreases with ${\text{Δ}}_{M}^{E}$ in the clustered network (see Fig. S14B for similar results in the uniform network).

What circuit mechanisms can explain the inverted-U relationship between tone discriminability and arousal in A1? Because this relationship is nonlinear, we reasoned that it may stem from a complex modulation of recurrent circuit dynamics. To investigate this, we modeled A1 as a recurrently-connected network of excitatory (E) and inhibitory (I) spiking neurons (Fig. 3A,B; Sec. IV B). Within this class of models, we compared alternative scenarios that differed in regard to two aspects: *(i)* the network architecture, and *(ii)* the implementation of arousal. By testing alternative models, we aimed to elucidate potential dynamical principles underlying the experimental observations.

We considered two different network architectures, which we refer to as “uniform” (Fig. 3A, Left) and “clustered” (Fig. 3B, Left). In the uniform model, neurons were connected randomly with homogeneous coupling strengths. In the clustered model, neurons were instead arranged into strongly-coupled clusters [36] (Sec. IV B 2), motivated by evidence of structural and functional assembly organization in cortical ensembles [37–46]. The two networks give rise to distinct dynamics: the uniform model generates asynchronous-irregular activity (Fig. 3A, Right), whereas the clustered model can generate metastable attractor dynamics [32–34, 36, 47–49] (Fig. 3B, Right). In the metastable regime, which occurs with strong intracluster coupling (Fig. S11), clusters spontaneously transition between states of high and low firing rate. Metastable activity has previously been shown to explain contextual modulations of stimulus processing and neural variability across a variety of settings [33, 34, 36, 48–54].

Experimental studies indicate that arousal-induced modulations of cortical activity are mediated by external projections from neuromodulatory systems (e.g., the cholinergic and noradrenergic systems) and thalamic pathways [1–3, 8]. Consistent with prior work [36, 48], here we aimed to capture the phenomenological effects of arousal by incorporating it as a modulation of the background (i.e., non-stimulus specific) input to the circuit (Figs. 3A,B). Because variations in pupil-linked arousal occur on slower timescales than stimulus-evoked neural responses [1, 14, 20, 55], such modulations were introduced as constant shifts in the level of background drive to a particular cell.

To constrain the nature of the arousal modulation in the network model, we quantified how spontaneous firing rates varied with pupil diameter in the experimental recordings (Sec. IV E). For cells that exhibited a monotonic trend between firing rate and pupil diameter, we observed comparable fractions of positive and negative rate modulations (Fig. 3C-E; Fig. S8 for individual sessions). This analysis indicates that arousal has heterogeneous impacts on spontaneous activity in A1, and can induce both increases and decreases in firing rate.

To capture this diversity of responses, we modeled arousal as a heterogeneous modulation of the background input to E cells (Sec. IV B 4). Namely, we considered a scenario in which, with increasing arousal, some E neurons received a larger background input, while other E neurons received a smaller background input; these modulations were performed in a spatially-random fashion and the average input across all E neurons was left unchanged (Fig. 3F,Left; inputs to I cells were not modulated). The strength of the modulation is controlled by a single parameter – the “input heterogeneity” $\left({\text{Δ}}_{H}^{E}\right)$ – which is proportional to the spread of the background input distribution. To compare against the data, we computed the fraction of neurons in the model whose spontaneous rates increased or decreased with ${\text{Δ}}_{H}^{E}$ (Sec. IV F). Similar to the experiments, single-cell responses were mixed (Fig. 3F, Right), with large proportions of cells exhibiting either enhanced or suppressed spontaneous rates with increasing ${\text{Δ}}_{H}^{E}$.

A natural alternative to the input heterogeneity model would be to implement arousal as a uniform increase in the background input to E cells (Fig. 3G,Left; Sec. IV B 4). However, this “input mean” modulation, parameterized by the quantity ${\text{Δ}}_{M}^{E}$, resulted in only positive rate modulations (Fig. 3G Right). We thus conclude that of the simple scenarios considered, the input heterogeneity model is the one that captures the diversity of arousal-related rate changes observed in the empirical data.

### C. The clustered model captures the inverted-U relationship between decoding performance and arousal

**FIG. 4.**
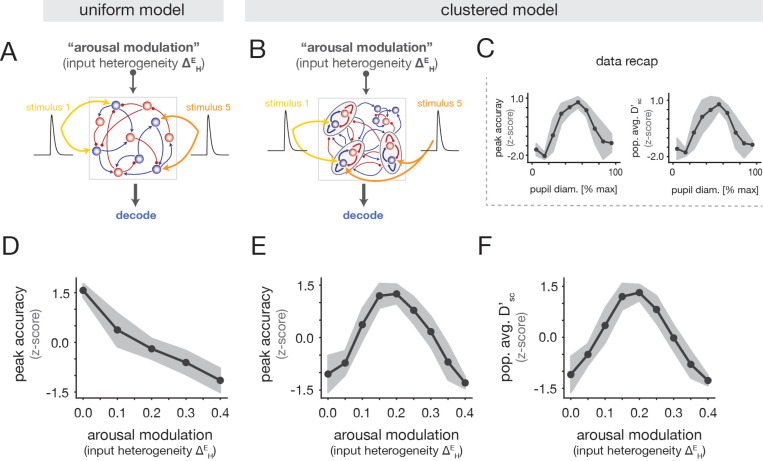
The clustered model captures the inverted-U relationship between decoding performance and arousal. **(A,B)** Schematics demonstrating the inclusion of sensory stimuli into the uniform and clustered network models (Sec. IV B 3). Each stimulus (five in total) was presented several times, and a linear decoder was trained to predict stimulus identity given activity from a random subsample of the E cells (Sec. IV C). **(C)** Recap of key findings from the experimental data. **Left**: Peak accuracy (z-scored) *vs.* pupil diameter. **Right**: Population-averaged $D_{\text{sc}}^{\prime }$ (z-scored) *vs.* pupil diameter. The two panels are reproduced from Figs. 2D and F; solid lines and shaded areas represent the mean ± 1 S.D. of the session-pooled data. **(D)** Peak accuracy (z-scored) *vs.* the ${\text{Δ}}_{H}^{E}$ arousal modulation in the uniform model. **(E)** Same as **(D)** but for the clustered model. **(F)** Population-averaged $D_{\text{sc}}^{\prime }$ (z-scored) *vs.* the ${\text{Δ}}_{H}^{E}$ arousal modulation in the clustered model. In panels **D-F**, solid lines and shaded areas indicate the mean ± 1 S.D. across ten simulations. See Fig. S15 for results without normalization.

We next examined whether the “inverted-U” relationship (see recap in Fig. 4C) could be reproduced in either circuit model (“uniform” or “clustered”) as a function of the input heterogeneity arousal modulation $\left({\text{Δ}}_{H}^{E}\right)$. To study stimulus coding in the network models, we modeled auditory stimuli as additional excitatory inputs that were localized to specific subgroups of E cells (Fig. 4A,B; Sec. IV B 3). In the clustered networks, a given stimulus targeted a randomly-chosen subset of the clusters, and in the uniform networks, each stimulus targeted a random subset of the E cells; the total number of stimulated cells was the same in both models. To match the experiments, we modeled five stimuli and allowed for overlap in the cell subgroups targeted by different stimuli, in line with the fact that cells could respond to multiple tones in the empirical data (Fig. S9).

As for the neural recordings, we trained a linear decoder to classify stimulus identity from single-trial population activity (Sec. IV C). The uniform and clustered networks exhibited distinct relationships between decoding performance and the ${\text{Δ}}_{H}^{E}$ arousal modulation. In the uniform model, decoding accuracy monotonically decreased with ${\text{Δ}}_{H}^{E}$ (Fig. 4D), inconsistent with the experimental data. By contrast, the clustered model exhibited optimal performance at intermediate values of ${\text{Δ}}_{H}^{E}$ (Fig. 4E), and reproduced the inverted-U relationship in the data (Fig. 4C). We note that an inverted-U also arose in clustered networks under strong increases of the mean input to E cells (Fig. S16A); but crucially, this alternative modulation fails to reproduce the heterogeneity of spontaneous rate changes with arousal (Fig. 3G), and drives the network to an unrealistic regime characterized by excessive activity (Fig. S16B). We also computed the single-cell discriminability index $\left(D_{\text{sc}}^{\prime }\right)$ as a function of the ${\text{Δ}}_{H}^{E}$ (Sec. IV G). We observed that the population-averaged $D_{\text{sc}}^{\prime }$ was maximal at an intermediate value of ${\text{Δ}}_{H}^{E}$ (Fig. 4F), matching the non-monotonic trend in the data (Fig. 4C).

Altogether, we conclude that the clustered architecture in conjunction with the input heterogeneity modulation can capture the observed inverted-U relationships between stimulus discriminability and arousal at both the population and single-cell levels. In the next section, we examine the network mechanism underlying these effects.

### D. The arousal modulation controls the dynamical regime of the clustered network model

Because the inverted-U relationship emerged in the clustered networks but was absent in the uniform model, we reasoned that it must rely on a modulation of the metastable dynamics that are unique to the clustered circuit. To investigate this, we used mean-field theory (MFT) to elucidate how the ${\text{Δ}}_{H}^{E}$ arousal modulation impacts spontaneous cluster dynamics (Sec. IV L). Although the MFT does not quantitatively describe the simulations (Sec. IV L 3), it provides useful qualitative insight.

**FIG. 5.**
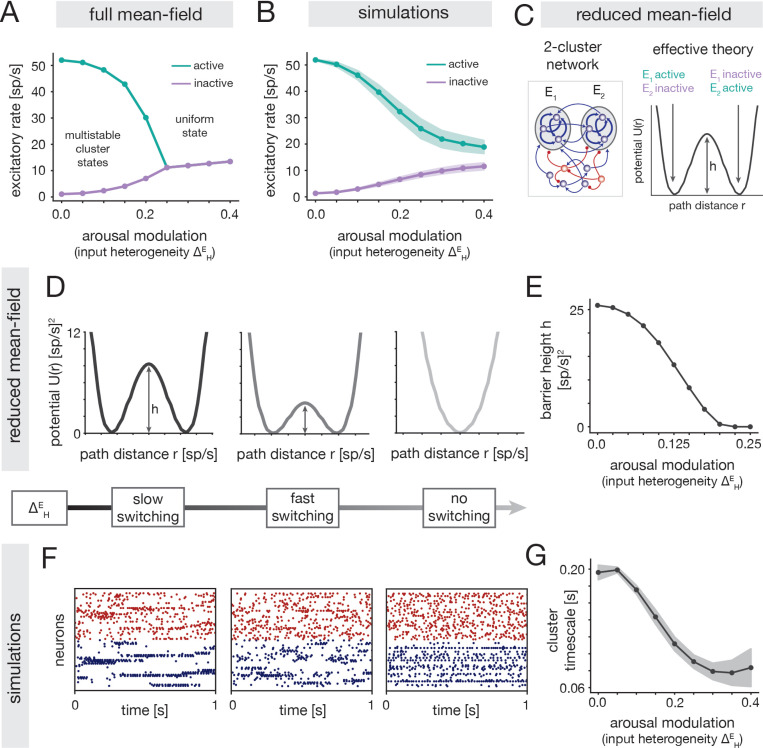
The arousal modulation controls the dynamical regime of the clustered network model. **(A)** Mean-field firing rates of active and inactive excitatory clusters as a function of the ${\text{Δ}}_{H}^{E}$ arousal modulation (Sec. IV L). We show results for the multistable cluster state with $n_{A}=3$ active clusters (Sec. IV L 4; see Fig. S12A for results with different $n_{A}$). Note that beyond a certain ${\text{Δ}}_{H}^{E}$, only the uniform solution exists. In these analyses, the mean-field calculations used a larger intracluster coupling than the simulations, so the comparison is only qualitative ( Sec. IV L 3). **(B)** Average firing rate of active and inactive excitatory clusters from simulations as a function of ${\text{Δ}}_{H}^{E}$ (Sec. IV H 2). We show the cluster rates conditioned on $n_{A}=3$ active clusters (Sec. IV H 2; see Fig. S12B for results with different $n_{A}$). **(C)** Schematic of the reduced mean-field analysis using a simplified network of two excitatory clusters. The behavior of the two clusters can be described via an effective potential energy, where the two wells correspond to the network’s two attractors (Sec. IV M; Fig. S13). **(D)** The effective potential of the 2-cluster network at three increasing values of ${\text{Δ}}_{H}^{E}$. **(E)** The barrier height h of the effective potential *vs.* ${\text{Δ}}_{H}^{E}$. (Note that the absolute range of ${\text{Δ}}_{H}^{E}$ values is not directly comparable between the reduced and full networks). **(F)** Example raster plots from simulations of the full clustered networks at three increasing values of ${\text{Δ}}_{H}^{E}$. **(G)** The average cluster activation timescale computed from simulations of the full clustered networks *vs.* ${\text{Δ}}_{H}^{E}$ (Sec. IV H 3). In panels B and G, solid lines and shaded areas show the mean ± 1 S.D. across ten network realizations.

At low ${\text{Δ}}_{H}^{E}$, MFT reveals the presence of multiple attractors, in which different subsets of the clusters are highly active (cluster states; Sec. IV L 4). Increasing ${\text{Δ}}_{H}^{E}$ decreases the firing rate of active clusters and increases the firing rate of inactive clusters, thus reducing the distinction between active and inactive states (Fig. 5A). Beyond a certain ${\text{Δ}}_{H}^{E}$, the theory predicts a transition from a multi-attractor to a single-attractor phase in which all clusters have the same moderate firing rate (uniform state). Network simulations qualitatively confirmed the intuitions from the MFT (Fig. 5B; Sec. IV H 2), though the sharp transition to a uniform state was softened in the simulations.

The arousal modulation also impacts the timescale of cluster switching dynamics. In order to theoretically elucidate the effect, we analyzed a reduced network composed of only two excitatory clusters (Fig. 5C Left; Sec. IV M). This network also displays cluster states, but has a simplified landscape with two attractors in which either cluster is active and the other inactive (Fig. S13). Using effective mean field theory [36, 48, 56], the attractors can be represented by two potential wells separated by a barrier (Fig. 5C Right; Sec. IV M). The height h of this barrier controls the rate of stochastic transitions between the two attractors, where larger barriers indicate slower switching and longer cluster activation periods [33, 48, 57].

The attractor landscape is significantly altered by the ${\text{Δ}}_{H}^{E}$ arousal modulation. For small ${\text{Δ}}_{H}^{E}$, the two wells are separated by a relatively large barrier, indicating inflexible dynamics with slow switching between attractors. At intermediate ${\text{Δ}}_{H}^{E}$ the two wells are preserved but the barrier height decreases (Fig. 5D Left, Middle), implying more flexible cluster dynamics with faster switching between states. For yet larger ${\text{Δ}}_{H}^{E}$, there is a transition from a 2-attractor phase to a single-attractor phase, wherein the two wells merge into a single well (Fig. 5D, Right); this transition indicates the loss of metastable cluster states. The theoretical insights from the reduced circuit were verified in simulations of the full clustered model, where we observed a shortening of cluster activation periods with increasing ${\text{Δ}}_{H}^{E}$ (Fig. 5F Left, Middle; Fig. 5G), consistent with the shrinking barrier in the reduced network (Fig. 5E). Visual inspection of network activity also revealed a degradation of metastable cluster states for large ${\text{Δ}}_{H}^{E}$ (Fig. 5F, Right), consistent with a transition to a near-uniform phase.

### E. Modulations of cluster dynamics underlie the inverted-U relationship in the network model

**FIG. 6.**
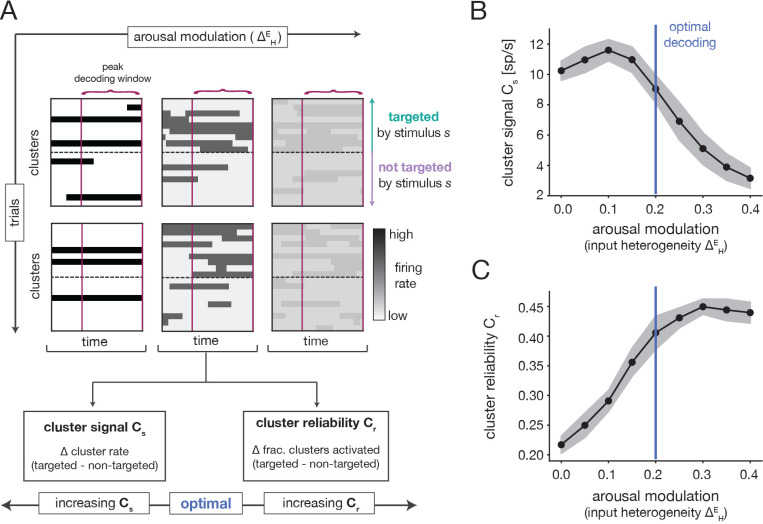
Modulations of cluster dynamics provide intuition for the inverted-U relationship. **(A)** Schematics demonstrating variations in single-trial evoked responses as a function of the ${\text{Δ}}_{H}^{E}$ arousal modulation in the clustered model. The rectangular panels illustrate cluster firing rates in response to a stimulus s (plotted relative to the time of peak decoding accuracy). At a given ${\text{Δ}}_{H}^{E}$, we compute two quantities to characterize the cluster activity pattern: the “cluster signal” $C_{s}$ and the “cluster reliability” $C_{r}$ (Sec. IV I). **(B)** The cluster signal as a function of ${\text{Δ}}_{H}^{E}$. **C** The cluster reliability as a function of ${\text{Δ}}_{H}^{E}$. Solid lines and shaded areas indicate the mean ± 1 S.D. across ten network realizations. The vertical blue lines indicates the value of ${\text{Δ}}_{H}^{E}$ where decoding performance is optimal (see Fig. 4E).

Since stimulus properties do not depend on the arousal modulation, any variations in stimulus processing with ${\text{Δ}}_{H}^{E}$ must be driven by changes in the spontaneous dynamics. We can thus use the insights of the previous section to develop intuition for the inverted-U nature of the decoding performance. To begin, we note that stimulus identity would be perfectly read-out from population activity if each stimulus could strongly activate all of its targeted clusters on every trial and strongly suppress all non-targeted clusters. To examine the extent to which this ideal scenario occurs, we quantified two properties of the cluster activation pattern in response to stimulus presentation (Fig. 6A): (i) the difference between the average firing-rates of targeted and non-targeted clusters (i.e., the “cluster signal” (Sec. IV I 1); and (ii) the difference between the fractions of targeted and non-targeted clusters that are activated (i.e., the “cluster reliability” (Sec. IV I 2).

The cluster signal increased slightly and then strongly decreased as a function of the ${\text{Δ}}_{H}^{E}$ arousal modulation (Fig. 6B). At low ${\text{Δ}}_{H}^{E}$, there is a large separation in the spontaneous firing rates of active and inactive clusters (Fig. 5A,B). Because stimulus presentation biases the activation of targeted clusters (Fig. S17A,B), the cluster signal is thus high in this regime (Fig. 6A Left). When ${\text{Δ}}_{H}^{E}$ is increased slightly, the contrast between active and inactive clusters remains large; at the same time, transitions between cluster states become easier and more frequent (Fig. 5D-G). This enables an increase in the relative amount of targeted cluster activation in response to a stimulus (Fig. S17C), which yields the small rise in the cluster signal. As ${\text{Δ}}_{H}^{E}$ is increased further, the spontaneous firing rates of active and inactive clusters converge (Fig. 5A,B). In consequence, the distinction between the evoked firing rates of targeted and non-targeted clusters also decreases, and the cluster signal falls off (Fig. 6A Right).

The cluster reliability exhibited the opposite trend as the cluster signal and increased with ${\text{Δ}}_{H}^{E}$ (Fig. 6C). For small ${\text{Δ}}_{H}^{E}$, spontaneous cluster dynamics are slow and inflexible (Fig. 5D-G) and only a fraction of all clusters activate in a fixed time window (Fig. S17D). Because stimuli are not strong enough to completely override the ongoing dynamics, the same is true during evoked dynamics. In consequence, only a fraction of all targeted clusters become activated in response to stimulation, and sometimes non-targeted clusters fail to deactivate (Fig. S17E). This results in inconsistent activation of targeted clusters and low cluster reliability (Fig. 6A Left). At intermediate ${\text{Δ}}_{H}^{E}$, cluster dynamics become faster and more malleable (Fig. 5D–G) and a larger fraction of clusters can spontaneously activate in a fixed time window (Fig. S17D). In consequence, stimuli can more dependably activate targeted clusters (Fig. S17B), and the the cluster reliability increases (Fig. 6A Middle). The slight increase in the cluster reliability at larger ${\text{Δ}}_{H}^{E}$ is driven by an overall increase in the number of clusters that transiently activate during the decoding window (Fig. S17D). However, it is difficult to estimate the reliability in this regime, because the boundary between activated and inactivated states is less well-defined.

The variations in the cluster signal and reliability together provide intuition for the inverted-U shape of the decoding performance (Fig. 4E). For intermediate ${\text{Δ}}_{H}^{E}$, both the signal and reliability are relatively high (Fig. 6B-C). In this optimal regime, the decoding performance is maximal. For both lower and higher ${\text{Δ}}_{H}^{E}$, either the reliability or signal drops significantly, leading to worse performance. The key insight is that the arousal modulation affects both the overall strength and consistency of cluster activation patterns, which combine to determine the efficacy with which stimuli are encoded.

### F. The clustered network model captures changes in neural variability with arousal

**FIG. 7.**
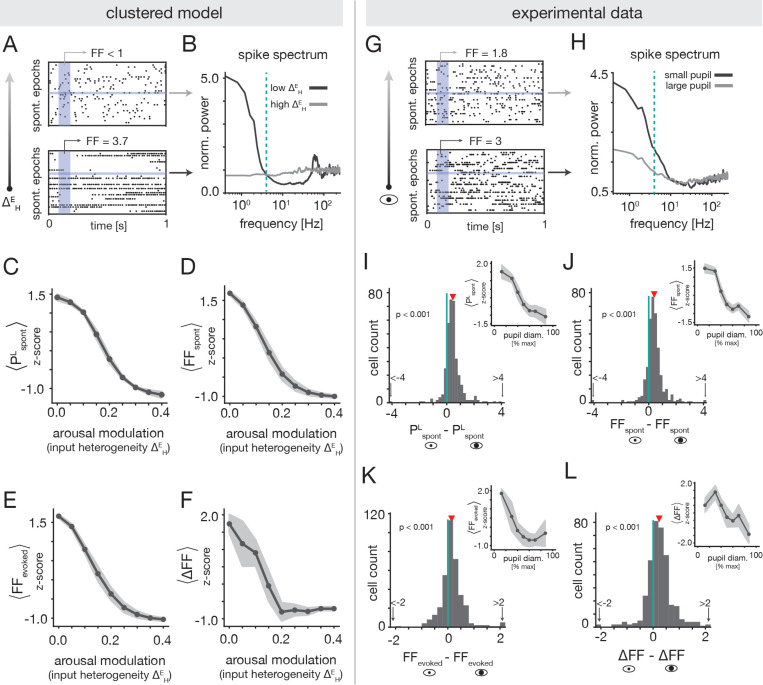
The clustered network captures changes in neural variability with arousal. **(A)** Spontaneous activity of a single neuron from the clustered model across several 1-second epochs. The ${\text{Δ}}_{H}^{E}$ arousal modulation is low in the bottom panel and high in the top panel. **(B)** Rate-normalized spike spectra of the neuron in panel **(A)** at low and high ${\text{Δ}}_{H}^{E}$. **(C)** The cell-averaged low-frequency power during spontaneous activity (z-scored) *vs.* ${\text{Δ}}_{H}^{E}$. **(D)** The cell-averaged spontaneous FF (z-scored) *vs.* ${\text{Δ}}_{H}^{E}$. **(E)** The cell-averaged evoked FF (z-scored) *vs.* ${\text{Δ}}_{H}^{E}$. **(F)** The cell-averaged spontaneous minus evoked FF (z-scored) *vs.* ${\text{Δ}}_{H}^{E}$. **(G)** Spontaneous activity of a single-unit from A1 across several 1-second epochs. The pupil diameter was small (< 25% of max dilation) in the bottom panel and large (> 75% of max dilation) in the top panel. **(H)** Rate normalized spike spectra of the unit in panel **(G)** for small and large pupil diameters. **(I)** Distribution of the difference in spontaneous low-frequency power $\left({\text{P}}_{\text{spont}}^{\text{L}}\right)$ between small (< 25% of max dilation) and large (> 75% of max dilation) pupil diameters; the histogram contains cells pooled across sessions that sampled a broad range of arousals. There is a significant reduction in ${\text{P}}_{\text{spont}}^{\text{L}}$ for large pupil diameters (Wilcoxon signed-rank test, *n* = 339 units, *p*-value < 0.001). **Inset:** The cell-averaged low-frequency power *vs.* pupil diameter. **(J)** Same as **(I)** but for the spontaneous FF $\left(\text{F}{\text{F}}_{\text{spont}}\right)$. There is a significant reduction in $\text{F}{\text{F}}_{\text{spont}}$ for large pupil diameters (Wilcoxon signed-rank test, *n* = 361 units, *p*-value < 0.001). **(K)** Same as **(I)** but for the evoked FF $\left(\text{F}{\text{F}}_{\text{evoked}}\right)$. There is a significant reduction in $\text{F}{\text{F}}_{\text{evoked}}$ for large pupil diameters (Wilcoxon signed-rank test, *p* < 0.001, *n* = 361 units). **(L)** Same as **(I)** but for the difference between spontaneous and evoked FFs (ΔFF). There is a significant reduction in ΔFF for large pupil diameters (Wilcoxon signed-rank test, *p* < 0.001, *n* = 361 units). For panels **C-F**: Solid lines and shaded areas indicate the mean ± 1 S.D. across 10 simulations. For panels **I-L**: Red markers indicate the distribution mean. In the insets, solid lines and shaded areas indicate the mean ± 1 S.D. across sessions. Only sessions that sampled a broad pupil range were included (9 sessions in panel **(I)** and 7 sessions in panels **(J-L)**). See Sec. IV J and Sec. IV K for details on the spectral and Fano factor analyses.

In the clustered model, the transition from a metastable attractor phase to a uniform phase underlies the inverted-U nature of the decoding performance. Importantly, this transition also results in specific predictions about how arousal should impact the variability of spiking activity, which we can test for in the experimental data. For low values of the ${\text{Δ}}_{H}^{E}$ arousal modulation, clusters slowly switch between active and inactive states. These dynamics produce slow rate fluctuations at the level of single-neuron activity (Fig. 7A bottom), which disappear as ${\text{Δ}}_{H}^{E}$ increases and activity becomes more homogeneous (Fig. 7B top). To quantify this change in the temporal structure of spontaneous activity, we estimated the amount of low-frequency power in the spike spectra of individual cells (Fig. 7B; Sec. IV J). As expected from visual inspection of neural activity, we found a strong reduction in spontaneous low frequency power $\left(P_{\text{spont}}^{L}\right)$ with increasing ${\text{Δ}}_{H}^{E}$ (Fig. 7E). The suppression of slow temporal fluctuations by the arousal modulation is accompanied by reductions in the trial-to-trial variability of neuronal spike counts as quantified by the Fano factor (FF; Sec. IV K 1). Indeed, we found that both the spontaneous FF ($\text{F}{\text{F}}_{\text{spont}}$; Fig. 7D) and the evoked FF ($\text{F}{\text{F}}_{\text{evoked}}$; Fig. 7E) monotonically decreased with ${\text{Δ}}_{H}^{E}$. The fact that $\text{F}{\text{F}}_{\text{spont}}$ and $\text{F}{\text{F}}_{\text{evoked}}$ behave similarly is a consequence of the evoked activity being strongly shaped by the spontaneous dynamics (Fig. 6A). That is, while stimulus presentation does bias the activation of targeted clusters, stimuli are not so strong as to be able to activate all of them together on every trial. In this way, the evoked dynamics inherit much of the intrinsic variability present in the spontaneous dynamics.

We next tested for the predictions of the clustered model in the experimental recordings. Fig. 7G shows activity from an example unit whose spontaneous low-frequency power and FF are substantially reduced during high arousal. To quantify how arousal impacts low-frequency fluctuations and trial-to-trial variability in general, we computed the change in $P_{\text{spont}}^{L}$, $\text{F}{\text{F}}_{\text{spont}}$, and $\text{F}{\text{F}}_{\text{evoked}}$ between low and high arousal states (Sec. IV J 2 and IV K 2). We found significant reductions in all three measures for high arousal (large pupil diameter; Fig. 7I-K). To further examine the pupil-dependence of these quantities, we computed the cell-averaged $P_{\text{spont}}^{L}$, $\text{F}{\text{F}}_{\text{spont}}$, and $\text{F}{\text{F}}_{\text{evoked}}$ as a function of pupil diameter within each session. At the session-average level, we observed that the low-frequency power and spontaneous FF clearly decreased with pupil diameter (insets of Fig. 7I,J). The evoked FF also decreased, but tended to plateau at moderate-to-large pupil sizes (Fig. 7K). As a whole, these findings are qualitatively consistent with the predictions of the clustered model, and support the conclusion that low-frequency fluctuations and across-trial variability generally decrease with arousal in A1.

Although we found overall reductions in both the spontaneous and evoked FF at large pupil diameters, the effect was weaker for the evoked condition. One reason for this might be that stimulus presentation itself reduces neural variability, which could make the effects of arousal less apparent in evoked conditions. To test for stimulus-induced quenching of variability, we computed the difference between the spontaneous and evoked FF $\left(\text{ΔFF}=\text{F}{\text{F}}_{\text{spont}}-\right.\left.\text{F}{\text{F}}_{\text{evoked}}\right)$, marginalized across all pupil diameters (Sec. IV K 2). Consistent with past reports [35], we observed a significant reduction in the FF in evoked conditions (Fig. S10). Clustered networks with metastable attractor dynamics were previously proposed to explain this phenomenon [32, 33], and we indeed observe clear stimulus-induced quenching of variability in the model at low ${\text{Δ}}_{H}^{E}$ (Fig. 7F).

We also tested for the presence of an interaction between arousal-induced and stimulus-induced quenching of variability. The clustered model indicates that ΔFF is larger for small values of ${\text{Δ}}_{H}^{E}$ (Fig. 7F), such that stimulus-related reductions in variability are strongest in the regime where spontaneous variability is largest. Given this prediction, we thus investigated whether ΔFF differed between low and high arousal states in the experimental data (Sec. IV K 2). We found a small but significant reduction in ΔFF for large pupil diameters (Fig. 7L). Moreover, we observed a roughly decreasing trend in the session-averaged ΔFF as a function of pupil diameter (Fig. 7L, inset). Though the effects are slight, these findings show that variability quenching may be arousal-dependent, which could potentially be explained by arousal-induced modulations of metastable assembly dynamics.

## III. DISCUSSION

We investigated potential network mechanisms governing the relationship between arousal and sound discriminability in auditory cortex. Our analysis resulted in three main conclusions: (1) In recordings from mouse A1 during passive listening, the ability to decode tone frequency from population activity followed an inverted-U relationship with pupil-linked arousal; (2) The inverted-U relationship can be explained by a clustered network model via modulations of metastable attractor dynamics, with optimal stimulus coding achieved near a transition in the dynamical regime of the network; (3) The clustered model predicts reductions in neural variability and stimulus-induced variability quenching with arousal, which were observed in the empirical data.

This study was motivated by results in both humans [18, 19, 58] and mice [20, 22] showing that performance on auditory tasks follows an “inverted-U” relationship with arousal [1, 7, 27]. Despite characterization at the behavioral level, the neural origins of the non-monotonic relationship between performance and arousal are incompletely understood. A previous study found that in mice trained on a sound detection task, neural correlates of the inverted-U relationship emerged in A1 (and medial geniculate nucleus) during passive listening [20]. Specifically, the authors reported reduced variability of spontaneous membrane potential dynamics and increased magnitude and reliability of evoked responses (in both whole-cell and multi-unit recordings) at moderate arousal. Our results indicate that an inverted-U relationship can also emerge in population-level neural representations pertinent to sound discrimination. Although we showed this in the context of passive listening, future work could attempt to more directly link arousal-induced modulations of A1 activity to performance on perceptual decision-making tasks [22].

Not all studies have reported non-monotonic relationships between evoked response properties and arousal in mouse A1. Of note, one investigation that analyzed calcium imaging recordings found that that arousal monotonically improved population coding of tones [59]. Several factors could contribute to across-study discrepancies, including differences in recording technique (e.g., electrophysiology *vs.* calcium imaging) or stimulus properties (e.g., tone duration). Follow-up efforts could further examine the conditions under which monotonic *vs.* non-monotonic relationships emerge. A number of studies have also quantified the effects of locomotion – typically a very high arousal state – on activity in mouse A1 [28–31]. These investigations show that movement tends to suppress sound-evoked responses, which is generally consistent with the right-most part of the inverted-U curve.

Arousal regulates sensory processing via several pathways, including neuromodulation by cholinergic and noradrenergic centers [1, 2, 8–12]. These neuromodulatory systems project to auditory cortex [16, 60–62], and changes in cholinergic and noradrenergic activity in A1 track fluctuations in pupil diameter [16, 17]. Several studies have also highlighted the central role of thalamocortical projections (possibly relaying neuromodulatory signals [13, 63]) in mediating the impacts of arousal on sensory areas [1–3, 8, 14, 64–66]. In our phenomenological network model, arousal effects were mediated by changes in background input to a local cortical circuit representing A1. Although the model is agnostic to specific arousal pathways, we constrained the nature of the arousal modulation by examining the impact of arousal on spontaneous firing rates in A1. Broadly consistent with previous studies [30, 31, 67], we found that arousal and locomotion have heterogeneous effects on spontaneous firing rates. The physiological source of this diversity is unclear. Our circuit model incorporated the impact of arousal in an effective manner meant to capture the presence of both positive and negative rate modulations in the data. That said, integrating more physiological realism into the arousal mechanism would be an important extension of the model.

We showed that the inverted-U relationship between arousal and sound discrminability can be recovered in a spiking model in which neurons are organized into strongly-coupled clusters representing functional assemblies [33, 34]. Aside from its ability to reproduce the inverted-U relationship, this model is motivated by evidence of clustered organization and/or functional assemblies in sensory areas. For example, simultaneous whole-cell recordings show evidence of strongly-connected neuronal subnetworks in both rodent visual [37–40] and somatosensory cortex [41]. Further studies with electron-microscopy have additionally revealed structural modules in a much larger network containing hundreds of cells [42]. Cells in strongly-coupled ensembles also exhibit similar responses to sensory stimuli [38, 40, 42], indicating that these assemblies may act as basic cortical processing units [68]. In A1 specifically, one calcium imaging study showed that the functional architecture of population activity is consistent with the presence of partially-overlapping and strongly connected subnetworks [44].

Unlike networks with uniform connectivity, the clustered model naturally generates metastable attractor dynamics [32, 33, 47, 49, 69], which were crucial for recovering the inverted-U relationship. These metastable dynamics are characterized by the transient activation of neural assemblies on subsecond timescales, and arise due to stochastic transitions between a multiplicity of attractors [32–34]. Metastable dynamics consistent with the clustered model have been used to explain several features of cortical dynamics and computation [47, 49, 69], including stimulus-induced quenching of neural variability [32, 33] or dimensionality [50], motor generation [51], and different aspects of context-dependent sensory processing [34, 36, 54, 70].

In auditory cortex, some analyses have suggested the presence of attractor-like assembly dynamics and metastable activity patterns. In particular, Bathellier et al. [45] found that evoked firing patterns in A1 populations were organized into a small number of discrete “response modes”, where each mode was a subgroup of cells co-activated by certain stimuli. Transitions between different response modes were abrupt, indicative of attractor-like dynamics, and different local populations contained modes that were activated by distinct sets of sounds. In this way, a given sound could be represented by a specific activation pattern of multiple local “response modes” [46], akin to the encoding of stimuli via a particular cluster activation pattern in our circuit model. Other studies in auditory cortex have observed transient, subsecond “packets” of elevated population activity that occur sporadically during spontaneous periods and that constrain stimulus responses [71, 72], as well as evidence of locally-clustered activity in more superficial layers [73]. These empirical findings are broadly reminiscent of the metastable activity underlying our model, but more spatially-distributed recordings and targeted perturbation studies are necessary to directly test for the presence of these dynamics in A1.

Previous modeling studies examined the response of clustered networks to relatively small external perturbations, leading to monotonic variations in stimulus processing efficacy [36, 48]. Our study builds on those efforts to explore a broader range of state (arousal) modulations, which was necessary to observe the non-monotonic variation in decoding performance. Underlying the inverted-U relationship is a shift in the dynamical regime of the network from a metastable attractor phase with a multiplicity of states to a single-attractor phase. At one extreme, clusters alternate between strongly active and inactive modes, but switching dynamics are slow and inflexible. At the other extreme, cluster states are abolished in favor of a uniform state. Our crucial finding was that stimulus discriminability is maximized between these two extremes, where stimulus responses are both relatively strong and reliable. This result is reminiscent of the idea that information processing capabilities in neural systems can sometimes be enhanced in the vicinity of phase transitions [74–80]. In the clustered model, the transition from the metastable to the uniform phase was realized by introducing quenched disorder in the background inputs to the network. The disorder-induced transition we observe here adds to recent theoretical work showing that modulations of quenched input enable rich dynamical phenomena in recurrent circuits by unlocking a repertoire of network phases [80].

The clustered model also predicts arousal-induced reductions of neural variability. We observed this effect in the empirical data, providing some support for the proposed mechanism. In the model, decreases in variability are driven by a suppression of slow rate fluctuations as the network transitions from the multistable to uniform phase with increasing arousal. This mechanism is also related to past work in which modulations of bistable up-down dynamics were used to explain changes in variability during attention [53] and across different brain states in anesthetized rats [81]. The clustered model additionally displays a decrease in stimulus-induced quenching of variability at high arousal. Although one in study in ferrets found that variability quenching was independent of pupil size [67], we found some evidence for a reduction of stimulus-induced quenching in high arousal states, as suggested by the model. Because of the ubiquity of stimulus-induced variability quenching [35], investigating the detailed features of its state-dependence could be an interesting direction for future study.

The network mechanism presented here is likely one of several that could explain the non-monotonic relationship between arousal and population coding accuracy. Indeed, one recent study proposed a circuit model in which an inverted-U relationship between arousal and performance is generated via a disinhibitory pathway involving two interneuron classes [58]; however, it remains unclear if that model is consistent with neural data and if it would also explain arousal-dependent effects on variability. An additional limitation of our model is that it does not include the large-scale tonotopic organization of A1 that is intermixed with more local “salt-and-pepper” organization [44, 82–84]. Incorporating this additional spatial structure could allow for capturing a greater diversity of experimentally-observed phenomena linking the spatiotemporal structure of spontaneous and evoked dynamics, cell-type-specific state modulations, and neural variability.

## IV. METHODS

### A. Experimental Procedures

#### 1. Subjects

All procedures were carried out with approval from the University of Oregon Institutional Animal Care and Use Committee. Wild-type animals (female and male mice, 8–15 weeks at time of surgery) were of C57BL/6J background purchased from Jackson Laboratory and bred in-house. Mice were kept on a reverse light cycle and had ad-libitum access to food and water.

#### 2. Surgical procedures

All surgical procedures were performed in an aseptic environment with mice under 1–2 isoflurane anesthesia, maintaining an oxygen flow rate of 1.5 L/min, and homeothermic maintenance at 36.5 degrees Celsius. Mice were administered systemic analgesia (Meloxicam SR: 4 mg/kg & Buprenorphine SR: 0.5 mg/kg, Wildlife Pharmaceuticals) and a fluid supplement (1 ml lactated ringer’s solution) subcutaneously. Fur was removed from the skull, and the skin was sterilized. To access auditory areas, the skin, connective tissue, and part of the right temporalis muscle were resected, and cleaned as necessary. A custom-designed headplate was affixed to the skull using dental cement (RelyX Unicem Aplicap, 3M) and covered with silicone elastomer (Kwik-sil, World Precision Instruments), and skin was affixed to the outside edge of the headpost as necessary (Vetbond, 3M). Mice were allowed to recover for three days in an incubator recovery chamber. A more detailed procedure can be found in [22, 85].

Mice were habituated to handling and head fixation for 2–3 days with increasing duration prior to craniotomy. This is a necessary step for well-being and also helps increase the likelihood that mice enter a broad range of arousal states across the wakefulness spectrum. The habituation of head-fixation atop a treadmill allowed mice to choose to locomote or remain still and quiescent. Craniotomy followed the same aseptic and analgesic procedures as mentioned above. Mice were anesthetized with isoflurane and affixed to the stereotax where a <1 mm circular craniotomy was drilled over the right auditory cortex (AP: −2.9 mm, LM: 4.4 mm, relative to bregma) with dura left intact. A small well was created surrounding the craniotomy with flowable composite (Flow-it, Pentron), and a piece of plastic was secured lateral to the well to act as a shield for the probe. The craniotomy was filled with silicone elastomer (Kwik-sil, World Precision Instruments) until the start of the recording session. Mice were allowed to recover overnight, and recovery was monitored.

#### 3. Extracellular recordings

On the day of a recording, a mouse was affixed onto a treadmill and the Kwik-sil was removed. The craniotomy was immediately filled with saline, and a high-density silicone probe (Neuropixels 1.0, imec) [86] was inserted perpendicular to the brain surface using a motorized micromanipulator (M225A, Sutter Instruments) at low speed (∼ 2–4 *μ*m/second) until all layers of the auditory cortex were covered (1.5–2.5 mm). After the Neuropixels probe reached a desired depth, the remaining saline was removed and the craniotomy was filled with 1% agarose mixture in saline and covered with mineral oil to keep the brain surface moist. A recording was started at least 20 minutes after the completion of probe insertion to ensure the stability of the probe and the brain. Recordings were made in up to 5 sessions from one mouse depending on the status of the brain surface. For the last recording session, the Neuropixels probe was covered with DiI (Vybrant solution, Thermofisher Scientific) for histology.

Neurophysiology data was acquired using the PXIe acquisition module (imec) in a NI PXIe-1071 chassis (National Instruments) and open-ephys software (OpenEphys) at gain of 250 (LFP), and 500 (APs). An output pulse from the OpenEphys software was manually toggled between 1 Hz and 10 Hz to give an accurate and discrete timestamp to the Power 1401 digitizer, which allowed for accurate alignment and further synchronization of the behavioral data. Neuropixels data was sampled at a rate of 30 kHz. The recorded data was pre-processed with common-average referencing [87], [88] sorted with Kilosort2 [89], and then manually curated with phy GUI (https://github.com/cortex-lab/phy). For manual curation, each cluster was compared with other clusters based on the spike waveforms and cross-correlation. The clusters with high similarity were mainly inspected to determine whether they should be merged. Then, the cluster was labeled as a good single unit, multi-units, or noise depending on the quality of the cluster assessed by waveform consistency, amplitude, cross-correlation, and inter-spike intervals. To determine if the good single units were within the auditory cortex, the depth from phy was referenced. Then, it was confirmed with DiI track spanning after histology. Sessions where timestamps were not able to be aligned were discarded.

#### 4. Auditory stimulus presentation and spontaneous periods

Auditory stimuli were delivered using custom LabView (National instrument) scripts. Tones were calibrated to 60 dB SPL and waveforms were generated (NI PXI-4461, National Instruments) at 200 kHz sampling rate, conditioned (ED1, Tucker Davis Technologies), and transduced by electrostatic speakers (ES1, Tucker Davis Technologies). Each experimental session consisted of alternating spontaneous and auditory stimulation blocks, repeated for up to 2 hours. During spontaneous blocks, neural activity was recorded in the absence of stimulus presentation; each block lasted for five minutes. A spontaneous block was followed by 25 minutes of auditory stimulation. This design enabled us to record substantial amounts of both spontaneous activity (∼ 20–25 minutes/session) and evoked activity (∼ 75–100 minutes/session). The stimulus set consisted of five pure tones (2, 4, 8, 16, or 32 kHz), which were randomly interleaved and sampled from a uniform distribution. Each tone lasted for 25 ms (cosine ramp-up) followed by a 775 ms inter stimulus interval (ISI).

#### 5. Behavioral measures acquisition and analysis

All data collection was conducted using custom LabView scripts. Mice were headfixed atop a cylindrical treadmill (15 cm diameter, 20 cm width) and allowed to freely locomote. Locomotion speed was calculated via a rotary encoder (Encoder Products CO.; 15T-01SF-2500NV1RPP-F03-S1) attached to the axle of the treadmill. Signals from the rotary encoder were continuously converted into cm/s in real-time using LabView software at a rate of 100 Hz, and data was recorded using a Power 1401 digitizer.

The face was lit using an infrared LED (Digi-Key TSHG8200, 830 nm) adjusted to achieve uniform illumination of the face and eye. Additionally, a white LED (RadioShack 5 mm 276–0017) was manually titrated to achieve a wide dynamic range of the pupil, ensuring it remained visible during full dilation. Pupil videos were collected from a camera (Grasshopper 3, FLIR) with a lens (Telecentric TEC-55, Computar) and near-IR Bandpass filter (BNB10–43, MidOpt) with FlyCapture software (FLIR). Frames were triggered at 30 Hz through a Power 1401 Digitizer (Cambridge Electronic Design), and camera exposure times were recorded at a rate of 25 kHz. Online pupillometry was performed using LabView software according to previously described methods [22], and post-hoc analysis was performed using custom python scripts. See “Processing of raw pupillometry data”.

#### 6. Histological Analysis

Following the last recording session, a mouse was anesthetized and perfused using phosphate buffer and 4% paraformaldehyde. Then, the brain was kept in 4% paraformaldehyde, cryo-sectioned (CM3050S, Leica) at 100 μm thickness, and DAPI-stained. Slides were imaged and DiI tracks were manually registered with the Franklin-Paxinos atlas [90].

#### 7. Additional unit selection criteria

After following the procedures described in Sec. IV A 3 to identify putative single units from the Neuropixels recordings, we applied some additional criteria for the final unit selection process. First, we discarded all clusters whose average firing rate across the duration of the recording was less than 0.25 spikes/second. The remaining criteria mainly involved further analysis of the spike template amplitudes of each cluster that was identified as “good” after performing the spike sorting and manual curation steps detailed above. Examining the behavior of the template amplitudes (output by Kilosort) for a given cluster across time can reveal potential issues with electrode drift and the general quality of the cluster. Our analysis was designed to search for two potential issues in the spike template amplitudes. First, we considered the shape of the amplitude distribution in a sliding time window, and in each window, we looked for signatures of multiple peaks occurring in the corresponding distribution. The presence of multiple peaks in the amplitude distribution computed from a short block of time is an indication that the particular cluster should not be marked as a well-isolated single unit. Second, we looked for cases when the amplitude appeared to drift towards or away from very low values (i.e., towards or away from the “noise floor”) over time. This scenario implies that the cluster was not stably-tracked across the recording, and could result in the cluster exhibiting firing rate drift unrelated to changes in behavioral state.

To determine if the distribution of template amplitudes in a short time segment was composed of two or more separate peaks, we examined the amplitude data in non-overlapping, 5-minute windows over the entire dataset. For each window, we used the ‘gaussian kde’ function from the ‘scipy.stats’ python package to estimate the probability density function (pdf) of the amplitude data via kernel density estimation with a Gaussian kernel. For each window, we then determined the locations (i.e., amplitude values) and heights of all peaks in the corresponding pdf. If the pdf from a given window had more than one peak, we computed two additional quantities. First, we computed the ratio of the height of the tallest peak to the height of the second tallest peak in the window; we refer to this quantity as the “peak height ratio”. Smaller peak height ratios tend to correspond to more even splits of the data between the two groups. Second, we computed the percent difference between the locations of the two highest peaks in a window. Larger percent differences between the peak locations correspond to more well-separated groups. After computing these quantities, we found the set of time windows for which the peak height ratio was less than or equal to ten and for which the percent difference between peak locations was greater than or equal to forty. These cut values were selected so as to find time windows for which there were two (or more) well-separated template amplitude ranges that each contributed substantially to the total amount of data in the window. If at least 10% of all time windows satisfied the above criteria, then the corresponding cluster was not used in subsequent analyses.

To determine if the template amplitude for a given cluster appeared to drift into or out of the “noise floor” over time, we first estimated the noise floor as the smallest template amplitude of the cluster across the whole recording. As above, we then considered the pdf of the amplitudes in 5-minute bins. First, we computed the percent difference between the location of the tallest peak in the pdf of a given window and the location of the noise floor. If this percent difference was less than or equal to fifteen, then the corresponding window was marked as having template amplitudes that were concentrated near the noise floor. For each window, we also determined the location (i.e., amplitude) of the tallest peak in the pdf. We then computed the smallest and largest of those amplitudes across all time windows, and computed the percent difference between the resulting two values. This quantity, which we refer to as the maximum peak location difference, provides information about the range of template amplitudes sampled across the recording. We removed a cluster from subsequent analyses if the following criteria were met: *(i)* more than 10% (but not all) of time windows either had template amplitudes concentrated near the noise floor or in the bulk, and (ii) the maximum peak location difference was greater than or equal to twenty-five. These cut values were chosen so as to try and isolate clusters with significant drift towards or away from low amplitude values. All analyses in the main text were performed after applying the unit selection procedures described in this section.

#### 8. Processing of raw pupillometry data

Raw pupil diameter traces were subject to three processing steps: *(1)* artifact removal, *(2)* smoothing, and *(3)* normalization. The pupil-tracking procedure is imperfect, which can lead to artifacts in the pupil diameter traces such as abrupt drops or spikes. To mitigate the effect of these artifacts, we performed both automated and manual cleaning of the pupil traces in each session. Automated artifact removal consisted of finding and discarding periods of time associated with unnaturally-sharp jumps in pupil diameter values between nearby time points. At each time point $t_{n}$ in the pupil trace, we compared the difference in pupil diameter (normalized to the maximum value across the trace) between $t_{n}$ and $t_{n}+0.5$ ms. If the absolute difference in the normalized pupil diameter between those times exceeded a threshold of 0.08, then we removed the pupil data within a time window starting 250 ms before $t_{n}$ and ending 500 ms after $t_{n}$. This automated procedure removed a large majority of pupil artifacts, but pupil traces were still manually inspected afterwards for outstanding abnormalities. Remaining problematic time windows were tabulated, and the corresponding pupil data was removed from those periods. Pupil traces were also smoothed after artifact removal for easier manipulation. This was achieved by taking a moving average of the pupil diameter timecourses using windows of length 1/30^th^ of a second sliding forward in 1 ms steps. Finally, the resulting pupil diameter trace of each session was re-normalized to the maximum value across the recording. Throughout the text, we display pupil diameters as a percentage of the maximum value (denoted as “% max”).

### B. Details of the circuit model

We modeled a local cortical circuit representing A1 as a recurrently-connected network of N spiking neurons, $N_{E}$ of which were excitatory (E) cells and $N_{I}$ of which were inhibitory (I) cells. Further details on the circuit modeling are provided below. All model parameters are shown in Table S1.

#### 1. Model of neuronal dynamics

Neuron activity evolved according to the leaky-integrate-and-fire (LIF) model with exponential excitatory and inhibitory synapses. In this model, the dynamics of the membrane potential of the $i^{th}$ neuron in population α∈{E,I} are described by

(1) $$\tau_{\text{m}}^{\alpha }\frac{dV_{i}^{\alpha }}{dt}=-V_{i}^{\alpha }+\tau_{\text{m}}^{\alpha }I_{\text{rec},i}^{\alpha }+\tau_{\text{m}}^{\alpha }I_{\text{b},i}^{\alpha }+\tau_{\text{m}}^{\alpha }I_{\text{stim},i}^{\alpha },$$

where $\tau_{\text{m}}^{\alpha }$ is the membrane time constant of cells in population α. $I_{\text{rec},i}^{\text{α}}$ is the recurrent input to cell i in population α from other neurons in the network, $I_{\text{b},i}^{\alpha }$ represents background external input, and $I_{\text{stim},i}^{\alpha }$ is an additional external input representing sensory stimulation. When the membrane potential $V_{i}^{\alpha }$ reaches a threshold $V_{\text{thresh}}^{\alpha }$, a spike is emitted by the neuron and its membrane potential is reset to a value $V_{\text{r}}^{\alpha }$. After spike emission, the membrane potential remains clamped at the reset value for a refractory period of length $\tau_{\text{ref}}^{\alpha }$.

The recurrent input is a sum of excitatory and inhibitory synaptic currents, such that $I_{\text{rec},i}^{\alpha }=I_{\text{rec},i}^{\alpha E}+I_{\text{rec},i}^{\alpha I}$. These currents obey the following differential equations:

(2) $$\tau_{\text{syn}}^{E}\frac{dI_{\text{rec},i}^{\alpha E}}{dt}=-I_{\text{rec},i}^{\alpha E}+\sum_{j=1}^{N_{E}}W_{ij}^{\alpha E}\sum_{k}\delta \left(t-t_{j}^{k,E}\right)$$

(3) $$\tau_{\text{syn}}^{I}\frac{dI_{\text{rec},i}^{\alpha I}}{dt}=-I_{\text{rec},i}^{\alpha I}+\sum_{j=1}^{N_{I}}W_{ij}^{\alpha I}\sum_{k}\delta \left(t-t_{j}^{k,I}\right).$$

In Eq. 3, $\tau_{\text{syn}}^{E}$ and $\tau_{\text{syn}}^{I}$ are the excitatory and inhibitory synaptic time constants, and $W_{ij}^{\alpha \beta }$ represents the strength of the synapse from the $j^{th}$ neuron of population β∈{E,I} to the $i^{th}$ neuron of population α; these weights depend on the network architecture (see Sec. IV B 2 below). Finally, $t_{j}^{k,\beta }$ is the time of the $k^{\text{th}}$ spike emitted by the $j^{th}$ neuron of population β.

In addition to the recurrent input, each neuron in population α received $C_{\text{ext}}^{\alpha E}$ connections from other excitatory cells outside of the local network. The background synaptic input at the $i^{th}$ neuron of population α evolved according to

(4) $$\tau_{\text{syn}}^{E}\frac{dI_{\text{b},i}^{\alpha }}{dt}=-I_{\text{b},i}^{\alpha }+J_{\text{ext}}^{\alpha E}\sum_{j=1}^{C_{\text{ext}}^{\alpha E}}\sum_{k}\delta \left(t-t_{ij}^{\alpha ,k}\right),$$

where ${\text{J}}_{\text{ext}}^{\alpha E}$ is the strength of external excitatory synapses to cells in population α, and where $t_{ij}^{\alpha ,k}$ is the $k^{\text{th}}$ spike time of the $j^{\text{th}}$ external cell targeting neuron i in population α. The spike times $t_{ij}^{\alpha ,k}$ were generated from a Poisson process with rate $\nu_{\text{ext},i}^{\alpha }$; spike trains were independent for each external synapse to a given cell, and there was no shared input across different cells. Under default conditions (i.e., no arousal modulation), $\nu_{\text{ext},i}^{\alpha }=\nu_{o}^{\alpha }\;\forall \;i$.

Finally, sensory stimuli were modeled as smoothly-varying, deterministic external inputs $I_{\text{stim},i}^{\alpha }(t)$ that directly entered the voltage equation of the corresponding neuron. Further details on the stimulus inputs are given in Sec. IV B 3.

#### 2. Recurrent network architectures

In the circuit model, the network architecture was either “uniform” or “clustered” (Fig. 3A,B). In the uniform case, neurons of type α∈{E,I} received a synaptic connection from $C^{\alpha \beta }=p^{\alpha \beta }N^{\beta }$ randomly chosen neurons of type β∈{E,I}. The weight of non-zero synaptic contacts from presynaptic neurons of type β to postsynaptic neurons of type α were set to $J^{\alpha \beta }$.

In the clustered model, excitatory and inhibitory neurons were arranged into p non-overlapping clusters. Each cluster contained $f^{\alpha }N^{\alpha }$ randomly chosen neurons of type α, and the remaining $\left(1-pf^{\alpha }\right)N^{\alpha }$ neurons were placed into an unclustered “background” population. Each neuron in a given cluster of type α received $f^{\beta }C^{\alpha \beta }$ connections from other neurons in the same cluster of type β, $\left(p-1\right)f^{\beta }C^{\alpha \beta }$ connections from neurons in different clusters of type β, and $\left(1-pf^{\beta }\right)C^{\alpha \beta }$ connections from neurons in the background population of type β. Each neuron in the background population of type α received $pf^{\beta }C^{\alpha \beta }$ connections from neurons in clusters of type β and $\left(1-pf^{\beta }\right)C^{\alpha \beta }$ connections from other neurons in the background population of type β. In this way, the total number of non-zero synaptic connections was the same for the uniform and clustered networks. The weights of non-zero synaptic connections between neurons in the same cluster, $J_{+}^{\alpha \beta }$, were generally stronger relative to the uniform case $\left(\left|J_{+}^{\alpha \beta }\right|>\left|J^{\alpha \beta }\right|\right)$. Moreover the weights of non-zero synaptic connections between neurons in different clusters, $J_{-}^{\alpha \beta }$, were generally weaker relative to the uniform case $\left(\left|J_{-}^{\alpha \beta }\right|<\left|J^{\alpha \beta }\right|\right)$. Synaptic contacts between cells in the background population and cells in the clusters were also weakened relative to the uniform model, and given by $J_{-}^{\alpha \beta }$. Finally, connection weights between background neurons were unchanged relative to the uniform architecture and equal to $J^{\alpha \beta }$.

The uniform and clustered networks were constructed such that the sum of all synaptic weights was the same for the two architectures. This was accomplished by fixing $J^{\alpha \beta }$ and $J_{+}^{\alpha \beta }$ and solving for the appropriate $J_{-}^{\alpha \beta }$. Following this procedure gives

(5) $$J_{-}^{\alpha \beta }=\frac{\left(f^{\alpha }+f^{\beta }-pf^{\alpha }f^{\beta }\right)J^{\alpha \beta }-f^{\alpha }f^{\beta }J_{+}^{\alpha \beta }}{f^{\alpha }+f^{\beta }-pf^{\alpha }f^{\beta }-f^{\alpha }f^{\beta }}.$$

#### 3. Sensory stimuli

To model stimulus-evoked activity, sensory signals were incorporated as additional, depolarizing external inputs to the cortical circuit (Eq. 1). For the clustered networks, 50% of the assemblies were chosen at random to receive input from a particular stimulus; for each selected cluster, stimulus-related input was applied to 50% of its E cells (chosen at random). In this way, two different stimuli in general targeted unique but overlapping sets of clusters. For the uniform networks, a given stimulus was modeled as an external input that was applied to a randomly-selected subset of the E cells; for each stimulus, the total number of stimulated neurons was chosen to be the same as in the clustered model. Throughout the text, we refer to cells and/or clusters that receive input from a particular stimulus s as “targeted” by that stimulus, and cells and/or clusters that do not receive input from stimulus s as “not-targeted” by that stimulus. Matching the five tones used in the experiments, we presented each model network with five different stimuli.

If the $i^{th}$ cell of population α∈{E,I} was targeted by a given stimulus, then the stimulus-related input to that cell took the form

(6) $$I_{\text{stim},i}^{\alpha }(t)=\left\{\begin{array}{ll} 0 & \text{if}\;t<t_{\text{stim}}\\ A_{\text{stim}}^{\alpha }\times \nu_{o}^{\alpha }C_{\text{ext}}^{\alpha E}J_{\text{ext}}^{\alpha E}\times s(t) & \text{if}\;t\geq t_{\text{stim}}; \end{array}\right.$$

otherwise, $I_{\text{stim},i}^{\alpha }(t)=0\;\forall t$. In Eq. 6, $t_{\text{stim}}$ is the onset time of the stimulus, $A_{\text{stim}}^{\alpha }\geq 0$ sets the amplitude of the stimulation signal for cells in population α, and s(t) describes the stimulus timecourse. Here, $A_{\text{stim}}^{I}=0$ since only E cells receive sensory stimulation. For the timecourse s(t), we used a difference of exponentials:

(7) $$s(t)=\gamma \left[e^{-\left(t-t_{\text{stim}}\right)/\tau_{d}}-e^{-\left(t-t_{\text{stim}}\right)/\tau_{r}}\right],$$

where $\gamma ={\left[{\left(\tau_{r}/\tau_{d}\right)}^{\frac{\tau_{r}}{\tau_{d}-\tau_{r}}}-{\left(\tau_{r}/\tau_{d}\right)}^{\frac{\tau_{d}}{\tau_{d}-\tau_{r}}}\right]}^{-1}$, $\tau_{r}$ is the rise time constant, and $\tau_{d}$ is the decay time constant.

#### 4. Arousal modulations

We modeled arousal as cell-type specific modifications of the background inputs to the recurrent circuit. Throughout the text, we refer to these modifications generally as “arousal modulations”. Here, we modeled scenarios where (i) the mean background input to E and/or I cells was uniformly increased (“input mean modulation”), or (ii) the background input to a given E and/or I cell was drawn from a Gaussian distribution with a fixed mean but increasing spread (“input heterogeneity modulation”).

For the input mean modulation, the rate of background external input to the $i^{th}$ cell in population α∈{E,I} was given by

(8) $$\nu_{\text{ext},i}^{\alpha }=\nu_{o}^{\alpha }+{\text{Δ}}_{M}^{\alpha }\nu_{o}^{\alpha },$$

where $\nu_{o}^{\alpha }$ is the baseline input rate to cells in population α (see Sec. IV B 1) and ${\text{Δ}}_{M}^{\alpha }\geq 0$ is a constant. Increasing ${\text{Δ}}_{M}^{\alpha }$ uniformly increases the background drive to all cells in population α. For the input heterogeneity modulation, the rate of background external input to the $i^{th}$ cell in population α was instead given by

(9) $$\nu_{\text{ext},i}^{\alpha }=\nu_{o}^{\alpha }+z_{i}{\text{Δ}}_{H}^{\alpha }\nu_{o}^{\alpha },$$

where $z_{i}$ is a standard Gaussian random variable and where ${\text{Δ}}_{H}^{\alpha }\geq 0$ is a constant. Increasing ${\text{Δ}}_{H}^{\alpha }$ increases the variance of the background input rates across the cells in population $\alpha \;\left[\mathrm{var}\left(\nu_{\text{ext}}^{\alpha }\right)={\left({\text{Δ}}_{H}^{\alpha }\nu_{o}^{\alpha }\right)}^{2}\right]$ while leaving the spatial average across cells approximately unchanged (inputs were not allowed to go negative). In other words, when ${\text{Δ}}_{H}^{\alpha }$ is non-zero, some cells in population α receive more input relative to baseline and others receive less input relative to baseline, but the average input across all cells in the population stays at the baseline value. The larger ${\text{Δ}}_{H}^{\alpha }$, the greater the heterogeneity of background inputs across the cell population. In the clustered networks, each assembly was subject to the same realization of the background input distribution; in this way, all clusters received the same amount of (spatially-averaged) input. In this study, we considered background input modulations that affected (i) the input heterogeneity of the excitatory population alone (i.e., ${\text{Δ}}_{H}^{E}\in [0,0.4]$ and ${\text{Δ}}_{H}^{I}=0$), or (ii) the mean input of the excitatory population alone (i.e., ${\text{Δ}}_{M}^{E}\in [0,0.4]$ and ${\text{Δ}}_{M}^{I}=0$).

#### 5. Numerical simulations

The dynamical system defined by Eqs. 1–4 was integrated using a discrete time step dt = 0.5 × 10^−4^ seconds. All spike times were forced to the simulation grid, and exact updates were performed between time steps. For each type of background input/arousal modulation (Sec. IV B 4), we performed simulations on several realizations of the clustered and/or uniform networks (5 realizations when ${\text{Δ}}_{M}^{E}$ was varied and 10 realizations when ${\text{Δ}}_{H}^{E}$ was varied). For the ${\text{Δ}}_{H}^{E}$ modulation, different network realizations were also associated with different realizations of the quenched disorder induced by the Gaussian random variable in Eq. 9. For most analyses, we simulated 30 trials of network activity per stimulus for each instance of the network architecture. For the Fano factor analyses of the clustered network model (Sec. IV K; Fig. 7C-F), we ran an additional set of simulations with a larger number of stimulus repetitions (200) per network realization. In all the simulations described thus far, each trial lasted 3.5 seconds and stimulus onset occurred at $t_{\text{stim}}=1$ second; the pre-stimulus period of each trial was considered “spontaneous” activity. We also ran an additional set of simulations for the power spectra analyses in the clustered model (Sec. IV J; Fig. 7B) in order to obtain longer continuous blocks of spontaneous activity. In this case, for each network realization, we simulated 30 trials of spontaneous-only activity (no stimulus presentation), where each trial lasted 2.7 seconds. In all simulations, different trials used different random initial conditions for neurons’ membrane potentials. All simulations of the network model were carried out in Python version 3.9.5.

### C. Population decoding analyses

Population decoding analyses assess the extent to which stimulus identity can be read-out from single-trial responses of a neural ensemble [91]. In the electrophysiological data, we used decoding techniques to examine how well tone frequency could be discriminated from population responses in auditory cortex. These analyses were performed either using all the available data within a session (i.e., without conditioning on arousal state; Fig. 2A; Fig. S3), or after parsing the data according to pupil-indexed arousal level with (Fig. 2B-E; Fig. S4; Fig. S6). In the model, we examined how decoding performance varied as a function of the ${\text{Δ}}_{H}^{E}$ arousal modulation in either uniform (Fig. 4D) or clustered networks (Fig. 4E), or as a function of the ${\text{Δ}}_{M}^{E}$ arousal modulation in the clustered networks (Fig. S16A). Below, we provide details on the decoding procedures applied to the electrophysiological data and the circuit models.

#### 1. Data selection procedure for decoding in the data

For analysis of the electrophysiological experiments, all good units (Sec. IV A 7) were used as features for the population decoding. Trials were defined as the time period spanning [−0.1,0.6] seconds relative to tone onset. To perform decoding of tone frequency without conditioning on arousal state, all evoked trials of a session – regardless of their pupil diameter – were gathered and considered for the analysis. To avoid biasing the decoder, we ensured that the number of trials per frequency was the same across all tones. If this wasn’t the case, we randomly subsampled the trials of each frequency to meet this criteria.

To quantify how arousal level impacts decoding performance, we parsed the trials in a given session according to pupil diameter. To begin, we computed the average pupil diameter across the pre-stimulus period of each evoked trial. We then split the trials into ten equally-sized partitions according to the deciles of the pre-stimulus pupil diameter distribution (Fig. S2); this partitioning procedure allowed us to use the maximum number of trials for the decoding analysis. Within each decile bin, we also randomly subsampled the trials to ensure that each partition contained the same number of trials per tone frequency. Subsequent decoding analyses were then performed independently for each pupil-based partition of the data. When examining the relationship between decoding performance and arousal in the absence of locomotion, trials with a treadmill velocity exceeding 1 cm/sec over the entire pre-stimulus period were excluded from the analysis.

#### 2. Data selection procedure for decoding in the circuit models

To decode stimulus identity in the circuit models, we randomly sampled a subset of excitatory cells to be used as features in the classification analysis. In the clustered networks, we drew one neuron from each cluster and one from the background population for a total of p+1=19 neurons/features. In the uniform networks, we drew p+1 excitatory neurons at random from the full population. We then averaged the decoding performance over 25 different runs, where each run used a different random sample of cells.

#### 3. Decoding stimulus identity as a function of time within trials

After gathering the relevant set of cells and trials for a particular decoding analysis, we trained a linear classifier to discriminate between stimuli given population activity from a particular time bin within a single trial. To this end, spikes from each cell were counted in a sliding window moving along the length of a trial. In the data, we used 100 ms time windows stepped forward in 10 ms increments; in the model, we used 100 ms windows stepped forward in 20 ms increments. Spike counts were computed in all relevant trials, yielding a large spike-count array of dimension $N_{\text{units}}\times N_{\text{trials}}\times N_{\text{windows}}$. Stimulus decoding was then performed separately on the data within each time bin.

Stimulus classification was carried out using version 0.24.2 of the scikit-learn Python package, and proceeded in several steps. Within a given time window, trials were split into training and testing sets. This was achieved using ten repetitions of stratified, 5-fold cross-validation. By using stratified folds, we ensured that the training and testing sets contained the same proportion of trials per stimulus. For each train-test split (50 in total), the training data was then used to fit a multiclass, linear discriminant classifier (‘sklearn.discriminant analysis.LinearDiscriminantAnalysis’with the ‘svd’ solver). Afterwards, the trained model was used to predict the stimulus identity of each trial in the test set.

To assess decoding performance, we examined the classification accuracy. Within a given time bin, the accuracy of a single train-test split was defined as the fraction of test trials whose stimulus identity was correctly predicted (for a single tone, it was the fraction of stimulus-specific test trials that were correctly predicted). The total, cross-validated accuracy of the time window was then computed as the average classification accuracy across all train-test splits. Repeating this process for each time bin yielded a time-course of decoding accuracy relative to stimulus onset (Fig. S3). The maximum of this time-course (i.e, the peak accuracy) was then computed to summarize the overall decoding performance (Fig. 2A, inset). Throughout the text, we refer to the time window corresponding to peak decoding accuracy as the “peak decoding window”.

#### 4. Significance of the overall decoding accuracy

To determine if tones could be decoded from A1 population activity using all the available trials (i.e., without conditioning on arousal state), we compared the true decoding accuracy to the distribution of accuracies obtained after random shufflings of the stimulus labels. For a given time window, we randomly permuted the tone frequency labels across trials, removing any association between population activity patterns and stimulus identity. In a stratified manner, we then randomly selected 80% of the label-shuffled trials for a training set, and used the remaining 20% for a test set. Using this train-test split, we followed the same classification procedure used for the un-shuffled data (Sec. IV C 3) to obtain one estimate of the null decoding accuracy. This process was then repeated for 100 random shufflings of the stimulus labels, yielding a distribution of null decoding accuracies for the given time bin. Finally, the true decoding accuracy in a given time bin was considered significantly above chance level if it was larger than the 95^th^ percentile of the null distribution. The peak decoding accuracy was well-above chance levels in all sessions (Fig. S3).

#### 5. Averaging decoding performance across experimental sessions and network simulations

In the arousal-conditioned decoding analysis, the peak accuracy was computed for each pupil diameter decile bin of a given session (Fig. 2C). To combine the results across recordings (Fig. 2D), we first standardized the ten accuracy values within a given session via z-score normalization. In this way, the normalized values indicate how far the decoding accuracy in a particular pupil decile deviates from the average accuracy across all pupil deciles. Each data point in a session (one per decile) was then binned according to it’s pupil diameter (i.e., the diameter at the middle of it’s decile). For this discretization, we used non-overlapping bins of width 10% of the maximum pupil diameter. If more than one data point from the same session fell within a single pupil diameter bin, we stored the average value of the normalized accuracy in that bin. This process was then repeated for each session, yielding a set of normalized accuracies in each pupil diameter bin (gray data points in Fig. 2D). Note that because different sessions explored different pupil dilation ranges, not all sessions contributed to every pupil diameter bin; specifically, there was more data at intermediate diameters relative to very small or large ones. To summarize how decoding performance varied with arousal, we computed the average normalized accuracy across sessions within each pupil diameter bin; the spread of the data was indicated by either a boxplot (Fig. 2D) or by the standard deviation (Fig. 4C) in each pupil diameter bin.

In the circuit models, the peak accuracy was computed separately at each value of the ${\text{Δ}}_{H}^{E}$ arousal modulation for a given network realization. Peak accuracies were z-scored within a realization, and the normalized values were then averaged across ten different simulations at each value of ${\text{Δ}}_{H}^{E}$ (Fig. 4D,E). Non-normalized versions of the decoding results are shown in Figs. S15A and B for the uniform and clustered networks, respectively. In the Supplement, we also show the peak accuracy as a function of the ${\text{Δ}}_{M}^{E}$ arousal modulation (average across 5 network realizations; Fig. S16).

#### 6. Comparing decoding performance between different pupil diameter conditions

To statistically quantify whether moderate arousal was associated with improvements in population-level decoding, we compared the peak decoding accuracy at moderate pupil diameters to the accuracy at either low or high diameters (Fig. 2E). For a given session, we first determined the pupil diameter decile bin that was centered closest to 50% of maximum pupil dilation. We then compared the accuracy in that central decile to the accuracy in either the first decile or last decile. Importantly, only a subset of recordings thoroughly sampled highly-constricted or highly-dilated pupil states (S2). For statistical comparison of moderate and low arousal conditions, we thus only considered sessions whose first pupil diameter decile was centered below 25% of maximum dilation (9 sessions total). Similarly, for comparing moderate and high arousal states, we considered sessions whose last pupil diameter decile was centered above 75% of maximum dilation (all 15 sessions met this criteria).

### D. Determining tone-responsiveness in the data

To determine if a cell responded significantly to a particular tone, we compared activity at a given time point in the 200 ms period after tone presentation (evoked period) to activity from the 200 ms period preceding tone onset (baseline period). To begin, trials were aligned to stimulus onset and grouped according to tone frequency; we denote the number of trials per tone as $N_{\text{trials}}$. For a given cell and tone, single-trial spike trains were binned in a 100 ms sliding window incremented in 1 ms steps. For each time bin ending in the evoked period, we compared the set of $N_{\text{trials}}$ spike counts in that bin to the set of $N_{\text{trials}}\times N_{\text{base bins}}$ spike counts from all baseline time bins (i.e., all bins that were fully contained in the pre-stimulus period). To determine whether activity in the evoked time bin was significantly different from baseline, we used the Mann-Whitney U test; p-values for each evoked time bin were corrected for the multiple comparisons in the evoked period using the Bonferroni correction. The tone response was considered significant in a given time bin if the corrected p-value was < 0.05, and a cell was considered responsive to the tone if the response was continuously significant for at least 5 ms during the evoked period.

### E. Quantifying relationships between single-unit spontaneous activity and arousal level in the data

To examine how spontaneous firing rates varied with arousal (Figs. 3C-E), we split the spontaneous periods of each experimental session into smaller windows of length 100 ms. For each window, we computed the spike count of every unit and the average pupil diameter over the window duration. Windows from all spontaneous periods were collected into a single dataset, and were then divided into ten groups according to the deciles of their pupil diameter distribution. For each decile bin, we computed (i) the average pupil diameter across all windows in the bin, and (ii) the average firing rate of each unit across all windows in the bin (see Figs. 3D,E for examples). Finally, we tested for a monotonic relationship between spontaneous firing rate and arousal by computing the Spearman correlation between a unit’s average firing rate in each pupil decile bin and the average pupil diameter in each decile bin. A correlation with *p* < 0.05 was considered statistically significant, and the sign of the correlation indicated whether the firing rate of the corresponding unit tended to increase (positive modulation) or decrease (negative modulation) with pupil diameter; non-significant correlations indicated the absence of a clear monotonic relationship between spontaneous firing rate and pupil diameter. Fig. 3C shows the fraction of units (averaged across sessions), with significant positive or negative correlations computed with this method. Results for individual sessions are shown in Fig. S8.

### F. Quantifying relationships between spontaneous activity and arousal modulations in the network models

To quantify how spontaneous activity was impacted by a given arousal modulation in the circuit models, we computed single-cell firing-rates in the absence of sensory stimuli. Specifically, for a fixed value of the arousal modulation (i.e. value of ${\text{Δ}}_{H}^{E}$ or ${\text{Δ}}_{M}^{E}$; Sec. IV B 4), rates of all cells were computed during the 800 ms window preceding stimulus onset in 150 trials (5 stimuli × 30 trials/stimulus) per network realization. We then averaged the spontaneous rates of each neuron across trials, and computed the Spearman correlation between the trial-averaged rate of each cell and the arousal modulation strength. A significant (*p* < 0.05) positive/negative correlation indicated a cell whose firing rate tended to monotonically increase/decrease with the arousal modulation. Figs. 3F and G show the fraction of all neurons in the clustered networks that exhibited significant positive or negative correlations with the ${\text{Δ}}_{H}^{E}$ or ${\text{Δ}}_{M}^{E}$ arousal modulations, respectively. Similar results for the unstructured networks are shown in Fig. S14.

### G. Single-cell discriminability

To examine neural discrminability on a single-cell level, we computed a standard metric for quantifying the separability between two stimulus response distributions. Given the responses of an individual cell to repeated presentations of two stimuli $s_{a}$ and $s_{b}$, the single-cell discriminability $\left(d^{\prime }\right)$ is:

(10) $$d^{\prime }\left(s_{a},s_{b}\right)=\frac{\left|\mu_{a}-\mu_{b}\right|}{\sqrt{\frac{1}{2}\left(\sigma_{a}^{2}+\sigma_{b}^{2}\right)}},$$

where $\mu_{a}$ and $\mu_{b}$ denote the average responses to the two stimuli, and where $\sigma_{a}$ and $\sigma_{b}$ denote the standard deviations of the two response distributions.

To compute an overall discriminability index in both the model and the data, we began by computing timecourses of the single-cell discriminability relative to stimulus presentation. To begin, all trials were aligned to stimulus onset. For each trial of a given stimulus, we computed binned spike counts of every cell in a sliding window (see subsections below for window parameters used in the model and data). In total, we obtained an array of spike counts (i.e., responses) of dimension $N_{\text{cells}}\times N_{\text{stimuli}}\times N_{\text{trials}}\times N_{\text{time bins}}$. In each time bin, the across-trial mean and standard deviation of the spike counts were used to compute $d^{\prime }$ for each cell and pair of stimuli, according to Eq. 10. To summarize the discriminability of an individual cell i in time bin t, we computed its average $d^{\prime }$ over all stimulus pairs, denoted here as ${\bar{d^{\prime }}}_{i,t}$. We then computed the average across all cells in each time bin, denoted as $\langle {\bar{d^{\prime }}}_{t}\rangle$. A final population-averaged discrminability index was defined as the maximum of the timecourse $\langle {\bar{d^{\prime }}}_{t}\rangle$; we denote this index as either the population-averaged $D_{sc}^{\prime }$ (or simply $\langle D_{\text{sc}}^{\prime }\rangle$). We also determined the time point t∗ at which $\langle {\bar{d^{\prime }}}_{t}\rangle$ was maximized, from which we computed an overall discrminability index for each cell i as $D_{\text{sc},i}^{\prime }={\bar{d^{\prime }}}_{i,t_{*}}$.

#### 1. Network model

To compute the single-cell discriminability in the clustered network model, spikes were binned using 100 ms windows incremented in 20 ms steps. For a given network realization, results were based off 30 trials per each of 5 stimuli. To summarize how the overall single-cell discriminability varied with the ${\text{Δ}}_{H}^{E}$ arousal modulation, we computed the population-averaged $D_{sc}^{\prime }\;\left(\langle D_{\text{sc}}^{\prime }\rangle \right)$ at each value of ${\text{Δ}}_{H}^{E}$ for a given network realization. We then z-scored $\langle D_{\text{sc}}^{\prime }\rangle$ across ${\text{Δ}}_{H}^{E}$, and computed the average of the normalized quantity over network realizations to obtain the final result in Fig. 4F (Fig. S15C shows results without z-score normalization).

#### 2. Experimental data

To compute the single-cell discriminability in the experimental data, tone trials were grouped according to the deciles of their pre-stimulus pupil diameter distribution, as described in Sec. IV C 1 for the population decoding analysis; each pupil-based partition (decile bin) was analyzed independently. After collecting the relevant subset of data, we computed binned spike counts of each cell in every trial using 100 ms windows incremented in 10 ms steps. We then followed the procedure above to compute the population-averaged $D_{sc}^{\prime }$ in each pupil decile bin of a session (see Fig. S7 for single-session results). To combine the population-averaged $D_{sc}^{\prime }$ across sessions (Fig. 2F) we used the method described in Sec. IV C 5 for the decoding performance.

To quantitatively test whether single-cell discrminability was improved at intermediate arousal relative to either low or high arousal, we compared the distributions of single-cell $D_{\text{sc}}^{\prime }$ values at different pupil diameters. First, we found the pupil decile bin that was centered closest to 50% of maximum dilation in each session. We also found the set of sessions whose first pupil decile bin was centered below 25% of maximum dilation (“low pupil sessions”, LS) and the set of sessions whose last pupil decile bin was centered above 75% of maximum dilation (“high pupil sessions”, HS). To compare $D_{\text{sc}}^{\prime }$ between low and middle pupil diameters, we pooled the single-cell $D_{\text{sc}}^{\prime }$ values from the first decile bin and central decile bin of each low pupil session into two groups: ${\left\{D_{\text{sc,low pupil}}^{\prime }\right\}}_{\text{LS}}$ and ${\left\{D_{\text{sc},\text{mid pupil}}^{\prime }\right\}}_{\text{LS}}$. To compare $D_{\text{sc}}^{\prime }$ between high and middle pupil diameters, we instead pooled the $D_{\text{sc}}^{\prime }$ values from the last decile bin and central decile bin of each high pupil session into two sets: ${\left\{D_{\text{sc},\text{high pupil}}^{\prime }\right\}}_{\text{HS}}$ and ${\left\{D_{\text{sc},\text{mid pupil}}^{\prime }\right\}}_{\text{HS}}$. We then compared ${\left\{D_{\text{sc},\text{low pupil}}^{\prime }\right\}}_{\text{LS}}$ and ${\left\{D_{\text{sc},\text{mid pupil}}^{\prime }\right\}}_{\text{LS}}$ (or ${\left\{D_{\text{sc, high pupil}}^{\prime }\right\}}_{\text{HS}}$ and ${\left\{D_{\text{sc},\text{mid pupil}}^{\prime }\right\}}_{\text{HS}}$ using paired statistical tests. In Fig. 2G, we show the distributions of the differences ${\left\{D_{\text{sc},\text{mid pupil}}^{\prime }-D_{\text{sc},\text{low pupil}}^{\prime }\right\}}_{\text{LS}}$ (top panel) and ${\left\{D_{\text{sc},\text{mid pupil}}^{\prime }-D_{\text{sc},\text{high pupil}}^{\prime }\right\}}_{\text{HS}}$ (bottom panel).

### H. Calculation of cluster rates and cluster timescale in the network model

#### 1. Time-varying cluster firing rates

To compute cluster firing rates in the clustered model, we first computed the time-dependent firing rate $r_{i}(t)$ of each neuron i by convolving its spike train with a Gaussian kernel of width σ=25 ms, incremented in 1 ms steps. The firing rate $r_{c}(t)$ of a given cluster c, was then computed as the average rate of its constituent neurons: $r_{c}(t)={\langle r_{i}(t)\rangle }_{i\in \text{cluster}\;c}$.

#### 2. Active and inactive cluster rates

To quantify how cluster activity varied with the ${\text{Δ}}_{H}^{E}$ arousal modulation (Fig. 5B) or intracluster coupling $J_{EE}^{+}$ (Fig. S11), we computed active and inactive cluster firing rates during the pre-stimulus period of simulated trials (here taken as the window spanning [−0.8, −0.1]s relative to stimulus onset). In a given trial, we first computed the time-dependent cluster firing rate $r_{c}(t)$ of every excitatory cluster (Sec. IV H 1). A cluster was considered “active” at time t if $r_{c}(t)\geq 15$ spks/sec. Given this criteria for cluster activation, we determined the number of active clusters $n_{A}$ as a function of time during the pre-stimulus period. By pooling across all time points with a particular value of $n_{A}$, we then calculated the probability of finding $n_{A}$ clusters active, as well as the average rate of active and inactive clusters as a function of $n_{A}$. We denote the trial-averaged active and inactive cluster firing rates as a function of $n_{A}$ as $r_{n_{A},↑}$ and $r_{n_{A},↓}$, respectively, and the trial-averaged probability of finding $n_{A}$ active clusters as $P\left(n_{A}\right)$. We determined the most likely number of active clusters, $n_{A}^{*}$, as the value corresponding to the maximum of the probability $P\left(n_{A}\right)$ (after averaging across network realizations).

For a fixed set of network parameters, only a few values of $n_{A}$ occurred with high likelihood (see Fig. S12C for the probability of finding $n_{A}$ active clusters at different values of the ${\text{Δ}}_{H}^{E}$ arousal modulation). For all values of the ${\text{Δ}}_{H}^{E}$ arousal modulation, the most likely number of active clusters was $n_{A}^{*}=3$. To summarize the behavior of the clustered networks as a function of ${\text{Δ}}_{H}^{E}$, we examined the active and inactive cluster rates conditioned on $n_{A}^{*}$ ($r_{n_{A}^{*},↑}$ and $r_{n_{A}^{*},↓}$, respectively; Fig. 5B). We also performed a supplementary analysis that examined the cluster rates for different values of $n_{A}$ (Fig. S12). Both of the aforementioned analyses were based on 150 trials per network realization (5 stimuli × 30 trials/stimulus). See Sec. IV L 3 and Fig. S11 for details on the analysis of active and inactive cluster rates as a function of the intracluster coupling $J_{EE}^{+}$.

#### 3. Cluster activation timescale

To calculate the average cluster activation timescale, we first used the threshold criteria in Sec. IV H 2 to determine the time points of cluster activation and inactivation during the pre-stimulus period of each trial (here taken as the window spanning [−0.8, −0.1]s relative to stimulus onset). The cluster timescale of a given trial was then calculated as the average duration across all cluster activation periods. For each network realization, we then averaged the timescale across 150 trials (5 stimuli × 30 trials/stimulus). Fig. 5G shows the average cluster activation timescale as a function of ${\text{Δ}}_{H}^{E}$.

### I. Analysis of evoked dynamics in the clustered network model

We characterized the evoked dynamics of the clustered networks using a number of quantities. In each case, we began by computing the time-dependent firing rate $r_{c}(t)$ of each excitatory cluster in every trial (Sec. IV H 1). To determine whether or not a cluster was active relative to its pre-stimulus activity, we computed a baseline-subtracted rate for each cluster, $g_{c}(t)$, by subtracting the time- and trial-averaged cluster rate during the 800 ms window preceding stimulus onset. A given cluster c was considered to be activated above baseline at time t if $g_{c}(t)$ exceeded a threshold of 1 spk/sec. All quantities below were computed over the 100 ms window that yielded peak decoding accuracy (i.e., the “peak decoding window”; Sec. IV C 3), and averaged over 150 trials per network realization (5 stimuli × 30 trials/stimulus).

#### 1. Cluster signal

To calculate the cluster signal ($C_{s}$; Fig. 6B), we began by computing the average, time-dependent firing rates of targeted and nontargeted clusters, $r_{\text{T}}(t)$ and $r_{\text{N}}(t)$, in every trial. We then computed the difference between the two average rates: $\text{Δ}r_{\text{T},\text{N}}(t)=r_{\text{T}}(t)-r_{\text{N}}(t)$. Finally, we averaged the difference $\text{Δ}r_{\text{T},\text{N}}(t)$ across the peak decoding window; this resulted in a single number $\text{Δ}r_{\text{T},\text{N}}^{*}$ for each trial of every stimulus. For a given network realization, the cluster signal was defined as the average of $\text{Δ}r_{\text{T},\text{N}}^{*}$ across all trials and stimuli.

#### 2. Cluster reliability

To compute the cluster reliability ($C_{r}$; Fig. 6C), we determined the fractions of targeted and nontargeted clusters, $f_{{\text{T}}_{↑}}$ and $f_{{\text{N}}_{↑}}$, that remained activated (relative to baseline) for at least 25 ms during the peak decoding window. We then computed the difference between those two fractions: $\text{Δ}f_{{\text{T}}_{↑},{\text{N}}_{↑}}=f_{{\text{T}}_{↑}}-f_{{\text{N}}_{↑}}$. For a given network realization, the cluster reliability was defined as the average of $\text{Δ}f_{{\text{T}}_{↑},{\text{N}}_{↑}}$ across all trials and stimuli. In the supplement, we also show the fraction of all clusters that remained activated above baseline for at least 25 ms during the peak decoding window ($f_{{\text{C}}_{↑}}$; Fig. S17D), as well as $f_{{\text{T}}_{↑}}$ and $f_{{\text{N}}_{↑}}$ separately (Fig. S17E).

#### 3. Additional measures

To quantify the probability that the active clusters at a given time were part of the stimulus-targeted subset, we computed the fraction $f_{↑\in \text{T}}(t)$ of active clusters that were part of the targeted subset at each time point in every trial. A single value for the pre-stimulus period, $f_{↑\in T}^{\text{spont}}$, was obtained by averaging $f_{↑\in \text{T}}$ over the 100 ms window preceding stimulus onset. This baseline value was compared to the average of $f_{↑\in \text{T}}(t)$ across the peak decoding window, denoted by $f_{↑\in T}^{\text{evoked}}$. For a given network realization, we then averaged $f_{↑\in T}^{\text{spont}}$ and $f_{↑\in T}^{\text{evoked}}$ across all trials and stimuli (Fig. S17A,B).

We also quantified the overall amount of time that targeted and nontargeted clusters were activated above baseline during the peak decoding window. To begin, we computed the fraction of the peak decoding window ${\tilde{\tau }}_{c_{↑}}$ for which each cluster c was active relative to baseline. The quantity ${\tilde{\tau }}_{c_{↑}}$ was then averaged across all targeted or nontargeted clusters, yielding two numbers, ${\tilde{\tau }}_{T_{↑}}$ or ${\tilde{\tau }}_{N_{↑}}$, respectively. To summarize the difference in the amount of targeted vs. nontargeted cluster activation, the quantity $\text{Δ}{\tilde{\tau }}_{N_{↑},T_{↑}}={\tilde{\tau }}_{T_{↑}}-{\tilde{\tau }}_{N_{↑}}$ was computed in each trial. For a given network realization, we then averaged $\text{Δ}{\tilde{\tau }}_{N_{↑},T_{↑}}$ across all trials and stimuli to obtain the final summary statistic (Fig. S17C).

### J. Spectral analyses

We utilized spectral analyses to characterize the temporal structure of spike trains during spontaneous periods in both the network model (Fig. 7B,C) and the experimental data (Fig. 7H,I). To compute the power spectrum of a neuronal spike train from a single trial (time window) of length T, we first binned the spike train at a fine temporal resolution of Δt=1 ms. The power spectrum of the binned spike train was then estimated using the multitaper method applied to point processes, as described in [92] and numerically-implemented in [93]. For the multitaper estimates, we used a time-bandwidth product of TW=5 and averaged over 2TW−1=9 tapers. The multitaper estimate of the spectrum from a given trial was then normalized by the average firing rate of the neuron across that trial; this rate-normalization is equivalent to normalizing the spectrum by that of a Poisson process with the same firing rate. Normalized spectra for a given neuron were then averaged across all trials of a particular condition to obtain a final, normalized power spectrum $S_{\text{norm}}(f)$. The low-frequency power was computed as the average of $S_{\text{norm}}(f)$ between 1–4 Hz.

#### 1. Network model

In the clustered network model, single-neuron spectra were estimated from several simulated trials of spontaneous activity conditioned on a particular value of the arousal modulation ${\text{Δ}}_{H}^{E}$. Specifically, for a given network realization and value of ${\text{Δ}}_{H}^{E}$, we used the method described above to compute the normalized spectrum $S_{\text{norm},i}(f)$ and the low-frequency power $P_{\text{spont},i}^{\text{L}}$ of cell i; these calculations were based on 30, 2.5 second trials of spontaneous activity. To summarize the overall extent of low-frequency fluctuations, we computed the average low-frequency power across all excitatory cells that had a firing rate of at least 1 spike/second for each value of ${\text{Δ}}_{H}^{E}$; we refer to this cell-averaged low-frequency power as $\langle P_{\text{spont}}^{\text{L}}\rangle$. Fig. 7C shows $\langle P_{\text{spont}}^{\text{L}}\rangle$ as function of the arousal modulation ${\text{Δ}}_{H}^{E}$.

#### 2. Experimental data

To compute power spectra in the neural data, the spontaneous blocks of each session were split into 2.5 second windows, and the average pupil diameter was computed across each one. The windows were then discretized into non-overlapping pupil diameter bins with upper boundaries located at [25%,35%,45%,55%,65%,75%,100%] of maximum dilation. This partitioning allowed us to evaluate changes in the spectra across a full range of arousal states and maintain a substantial number of trials in each pupil diameter bin for several of the sessions. To account for the uneven sampling of different pupil diameters within a given session, we subsampled the data such that all pupil bins in a session contained the same number of windows; results were then averaged across 50 different subsamplings. In total, 9 sessions exhibited a broad range of arousal states with at least 2 windows per pupil diameter bin.

For a given pupil diameter bin, we followed the procedure above to compute the normalized spectrum $S_{\text{norm},i}(f)$ and low-frequency power $P_{\text{spont},i}^{\text{L}}$ of each unit i in a session. To test for changes in low-frequency power between low and high arousal states, we pooled the single-unit $P_{\text{spont}}^{\text{L}}$ values from the first and last pupil bin across all sessions that sampled a broad range of pupil diameters, yielding two groups of values: $\left\{P_{\text{spont, low pupil}}^{L}\right\}$ and $\left\{P_{\text{spont, high pupil}}^{L}\right\}$. We then compared the groups using a paired statistical test, and visualized results by plotting the distribution of the difference $\left\{P_{\text{spont, low pupil}}^{L}-P_{\text{spont, high pupil}}^{L}\right\}$ (Fig. 7I). For each session, we also computed the cell-averaged low-frequency power, $\langle P_{\text{spont}}^{L}\rangle$, in each pupil diameter bin. To combine results across recordings, we z-score normalized $\langle P_{\text{spont}}^{L}\rangle$ across pupil bins within each session, and averaged the normalized values across sessions in each pupil diameter bin (Fig. 7I, inset). For these analyses, we only included cells that responded to at least one tone, had a spontaneous firing rate of at least 1 spike/second in all pupil diameter bins, and that had a non-zero spike count in all sampled time windows.

### K. Fano factor analyses

We used the Fano factor to characterize single-cell spiking variability in both the network model and the experimental data. For a given cell, the Fano factor (FF) is defined as

(11) $$FF=\frac{\mathrm{var}\left[n_{sp}\right]}{\langle n_{sp}\rangle },$$

where $n_{\text{sp}}$ indicates the spike count of the cell within a fixed time window, and where var[⋅] and $\langle \cdot \rangle$ indicate the variance and mean across repeated trials (or observation windows), respectively. In both the model and the data, we computed the FF during both spontaneous and evoked conditions.

#### 1. Network model

In the clustered network model, FFs were computed across 200 trials of a single stimulus for each network realization at a fixed value of the ${\text{Δ}}_{H}^{E}$ arousal modulation (see Sec. IV B 5 for details on the simulations). For this analysis, we focused on cells in stimulated clusters, excluding those that had a low spontaneous rate of < 1 spike/second at any ${\text{Δ}}_{H}^{E}$. To compute the FF of cell i, we binned the spikes in each trial using a 100 ms window incremented in 20 ms steps. The FF was then computed in each time bin (up to 200 ms after stimulus onset) according to Eq. 11, yielding a time course $FF_{i}(t)$. The spontaneous FF of cell $i\;\left(FF_{\text{spont},i}\right)$ was defined as the value of $FF_{i}(t)$ in the bin immediately preceding stimulus onset. To summarize the evoked FF, we first averaged $FF_{i}(t)$ across cells and determined the time point $t_{\text{F}{\text{F}}_{\min}}$ corresponding to the minimum of the population-averaged trace. For each cell i, the evoked FF $\left(FF_{\text{evoked},i}\right)$ was then defined as the value of $FF_{i}(t)$ at the time $t_{\text{F}{\text{F}}_{\min}}$. For each cell, we also computed the difference between the spontaneous and evoked FFs: $\text{Δ}FF_{i}=FF_{\text{spont},i}-FF_{\text{evoked},i}$. To summarize the results, we averaged each quantity across neurons; we refer to these population-averaged values as $\langle FF_{\text{spont}}\rangle$, $\langle FF_{\text{evoked}}\rangle$, and $\langle \text{Δ}FF\rangle$. Figs. 7D-F show $\langle FF_{\text{spont}}\rangle$, $\langle FF_{\text{evoked}}\rangle$, and $\langle \text{Δ}FF\rangle$, respectively, as a function of the ${\text{Δ}}_{H}^{E}$ arousal modulation.

#### 2. Experimental data

To compute spontaneous FFs as a function of arousal, the spontaneous blocks of each session were divided into 100 ms windows. Windows were then binned by average pupil diameter, using the same bins as the for the spectral analysis (Sec. IV J 2). To compute evoked FFs as a function of arousal, we parsed tone trials according to the average pupil diameter across the 100 ms window preceding stimulus onset, using the same pupil diameter bins as for the spontaneous data. This procedure ensured that spontaneous and evoked Fano factors were evaluated across similar pupil dilation ranges. To account for the differing numbers of windows and trials across pupil bins, we subsampled the data such that all pupil bins contained the same number of windows and trials per tone. In total, there were 7 sessions that thoroughly sampled a broad range of arousal states, defined as having at least 25 windows per pupil diameter bin in the spontaneous condition and at least 25 trials per pupil diameter bin and tone in the evoked condition.

For the spontaneous data, single-unit spike counts were computed in each window within a given pupil-based partition. The FF of each cell i was then computed via Eq. 11, and a final estimate of the spontaneous Fano factor, $FF_{\text{spont},i}$, was obtained by averaging across 100 random subsamples of the data. For the evoked FF, trials were first aligned to stimulus onset. In each trial, spikes from each cell were binned using 100 ms windows incremented in 1 ms steps. Using the trials for a given tone and pupil partition, FFs were calculated in each time bin (up to 200 ms after stimulus onset) according to Eq. 11, and results were averaged across 100 random subsamples of the data. This process yielded a time course $FF_{i,s}(t)$ for each cell i and stimulus s. To summarize evoked FFs, we first averaged the FF time courses for a particular stimulus s across the tone-responsive cells (Sec. IV D) and determined the time point $t_{\text{F}{\text{F}}_{\mathrm{s, min}}}$ corresponding to the minimum of the average trace. The evoked FF of cell i for stimulus s $\left(FF_{\text{evoked},i,s}\right)$ was then defined as the value of $FF_{i,s}(t)$ at the time point $t_{\text{F}{\text{F}}_{\mathrm{s, min}}}$. Finally, we obtained a summary statistic $FF_{\text{evoked},i}$ by averaging $FF_{\text{evoked},i,s}$ across all tones that induced a significant response in cell i. In each pupil bin, we also computed the difference $\text{Δ}FF_{i}$ between the spontaneous and evoked FFs of cell i: $\text{Δ}FF_{i}=FF_{\text{spont},i}-FF_{\text{evoked},i}$. Only cells that responded to at least one tone and that had an average spontaneous rate of ≥ 1 spk/second in every pupil bin were included in the analyses.

To test for a difference in the spontaneous FF between low and high arousal states, we pooled the single-unit $FF_{\text{spont}}$ values from the first and last pupil bin across all sessions that sampled a broad range of pupil diameters, yielding two groups of data: $\left\{FF_{\text{spont, low pupil}}\right\}$ and $\left\{FF_{\text{spont, high pupil}}\right\}$. We then compared the two groups with a paired statistical test, and visualized results by plotting the pooled distribution of the difference between low and high pupil states: $\left\{FF_{\text{spont, low pupil}}-FF_{\text{spont, high pupil}}\right\}$(Fig. 7J). The same procedure was also used to compare $FF_{\text{evoked}}$ and ΔFF between low and high arousal states (Figs. 7K,L). To examine session-average trends in $FF_{\text{spont}}$, $FF_{\text{evoked}}$, and ΔFF as a function of pupil diameter, we first averaged each measure across all relevant units in a session (see above). For a given session, this step yielded a cell-averaged spontaneous $\text{FF}\;\left(\langle FF_{\text{spont}}\rangle \right)$, evoked $\text{FF}\;\left(\langle FF_{\text{evoked}}\rangle \right)$, and difference between spontaneous and evoked $\text{FFs}\;(\langle \text{Δ}FF\rangle )$ in each pupil diameter bin. Within a given session, we z-score normalized $\langle FF_{\text{spont}}\rangle$, $\langle FF_{\text{evoked}}\rangle$, and $\langle \text{Δ}FF\rangle$ across pupil diameter bins, and then averaged the normalized values within each pupil diameter bin across sessions (Fig. 7J-L, insets).

To test for overall decreases in neural variability during stimulus presentation relative to spontaneous conditions, we marginalized the data in a session across all pupil diameters. Specifically, we combined the evoked trials or spontaneous windows from each pupil diameter bin (see above) into two aggregate datasets. Using the aggregate datasets, we then followed the methods described above to compute (i) a pupil-aggregated spontaneous Fano factor $FF_{\text{spont},i}$ of each cell i, and (ii) a pupil-aggregated evoked Fano factor $FF_{\text{evoked},i}$ of each cell i. Only cells that responded to at least one tone and that had an average spontaneous rate of ≥ 1 spk/second were included in the analysis. To test for stimulus-induced variability quenching, we pooled the single-unit $FF_{\text{spont}}$ and $FF_{\text{evoked}}$ values across all sessions that thoroughly sampled a broad pupil range (see above) to obtain two groups of data: $\left\{FF_{\text{evoked}}\right\}$ and $\left\{FF_{\text{spont}}\right\}$. We then compared $\left\{FF_{\text{evoked}}\right\}$ and $\left\{FF_{\text{spont}}\right\}$ using a paired statistical test (Fig. S10A). We also compared the cell-averaged spontaneous $\langle FF_{\text{spont}}\rangle$ and evoked $\langle FF_{\text{evoked}}\rangle$ in each session (Fig. S10B).

### L. Mean-field analyses on full clustered networks

To obtain theoretical insight into the effects of the ${\text{Δ}}_{H}^{E}$ arousal modulation on network activity, we performed a series of mean-field analyses for the clustered model. Mean-field theory is a commonly-applied technique for studying the collective dynamics of large, recurrently-connected networks of integrate-and-fire neurons [94], and has previously been used to study attractor dynamics in networks of LIF neurons with clusters [34, 36, 48, 95]. In what follows, we first explain the mean-field analysis carried out for the full clustered networks with both excitatory (E) and inhibitory (I) assemblies (associated with Fig. 5A of the main text). We then describe the effective mean-field theory performed on the reduced 2-cluster network (associated with Figs. 5C-E of the main text). Because observed changes in stimulus processing result only from changes in network dynamics induced by ${\text{Δ}}_{H}^{E}$ (versus from changes in the stimuli themselves), all mean-field analyses were performed for the “spontaneous” condition (i.e., in the absence of sensory stimulation).

Consider a network of LIF neurons composed of p E clusters, p I clusters, 1 “background” (unclustered) E population, and 1 “background” I population, for a total of 2(p+1) populations. We label the populations with a pair of superscripts (α,γ). The first superscript α∈{E,I} labels populations as excitatory or inhibitory, and the second superscript γ∈{1,…,p+1} specifies the population number, where the first p indices correspond to the cluster labels and the p+1 index corresponds to the background population. All neurons within the same population described by a given (α,γ) pair are assumed to have the same intrinsic parameters and receive exactly the same number and types of recurrent connections; the parameters describing the synaptic connectivity within and between each population type are given in Sec. IV B 2.

#### 1. No quenched randomness

To begin, we consider the scenario in which there is no arousal modulation acting on the network (i.e., ${\text{Δ}}_{H}^{\alpha }=0$ for α∈{E,I}). In this case, there is no quenched randomness in the external currents and the statistics of the inputs to cells in the same population are identical. Under these conditions, all neurons in population (α,γ) will share the same average firing rate, $\nu^{\alpha ,\gamma }$.

To perform the mean-field analysis – and arrive at an equation describing the average rates – one makes a set of assumptions about the operating regime of the network. The analysis proceeds by assuming that each neuron receives a large number of uncorrelated inputs, that the input and output spike trains received and emitted by cells in the network are independent, stationary Poisson processes, and that individual spikes from a presynaptic neuron induce only a small change in the voltage of a postsynpatic neuron relative to it’s firing threshold [94]. Under these conditions, one can make the diffusion approximation and replace the presynaptic input to population (α,γ) by a Gaussian white noise with mean $\mu^{\alpha ,\gamma }$ and standard deviation $\sigma^{\alpha ,\gamma }$. Assuming exponentially-decaying synapses with time constant $\tau_{s}$, the dynamics of a neuron i in population (α,γ) becomes

(12) $$\tau_{m}^{\alpha }\frac{dV_{i}^{\alpha ,\gamma }}{dt}=-V_{i}^{\alpha ,\gamma }(t)+\tau_{m}^{\alpha }I_{i}^{\alpha ,\gamma }(t)$$

(13) $$\tau_{s}\frac{dI_{i}^{\alpha ,\gamma }}{dt}=-I_{i}^{\alpha ,\gamma }(t)+\mu^{\alpha ,\gamma }+\sigma^{\alpha ,\gamma }\eta_{i}(t)$$

where $\tau_{m}^{\alpha }$ is the membrane time constant of neurons in population α, $V_{i}^{\alpha ,\gamma }$ is the membrane potential, $I_{i}^{\alpha ,\gamma }(t)$ is the total synaptic input from both external and recurrent sources, and where $\eta_{i}(t)$ is a Guassian white noise obeying $\langle \eta_{i}(t)\rangle =0$ and $\langle \eta_{i}(t)\eta_{i}\left(t^{\prime }\right)\rangle =\delta \left(t-t^{\prime }\right)$. The mean $\mu^{\alpha ,\gamma }$ and variance ${\left(\sigma^{\alpha ,\gamma }\right)}^{2}$ of the input depend on the network architecture. For the clustered networks studied here, we have

(14) $$\mu^{\alpha ,\gamma }=\left\{\begin{array}{l} \sum_{\beta =E,I}C^{\alpha \beta }f^{\beta }J_{+}^{\alpha \beta }\nu^{\beta ,\gamma }+\sum_{\beta =E,I}C^{\alpha \beta }f^{\beta }J_{-}^{\alpha \beta }\sum_{\begin{array}{c} \lambda =1\\ \lambda \neq \gamma \end{array}}^{p}\nu^{\beta ,\lambda }+\sum_{\beta =E,I}\left(1-pf^{\beta }\right)C^{\alpha \beta }J_{-}^{\alpha \beta }\nu^{\beta ,p+1}\\ +C_{\text{ext}}^{\alpha E}J_{\text{ext}}^{\alpha E}\nu_{o}^{\alpha },\;\text{if}\;\gamma =[1,\ldots ,p]\\ \sum_{\beta =E,I}C^{\alpha \beta }f^{\beta }J_{-}^{\alpha \beta }\sum_{\lambda =1}^{p}\nu^{\beta ,\lambda }+\sum_{\beta =E,I}\left(1-pf^{\beta }\right)C^{\alpha \beta }J^{\alpha \beta }\nu^{\beta ,p+1}+C_{\text{ext}}^{\alpha E}J_{\text{ext}}^{\alpha E}\nu_{o}^{\alpha },\;\text{if}\;\gamma =p+1 \end{array}\right.$$

and

(15) $${\left(\sigma^{\alpha ,\gamma }\right)}^{2}=\left\{\begin{array}{l} \sum_{\beta =E,I}C^{\alpha \beta }f^{\beta }{\left(J_{+}^{\alpha \beta }\right)}^{2}\nu^{\beta ,\gamma }+\sum_{\beta =E,I}C^{\alpha \beta }f^{\beta }{\left(J_{-}^{\alpha \beta }\right)}^{2}\sum_{\begin{array}{c} \lambda =1\\ \lambda \neq \gamma \end{array}}^{p}\nu^{\beta ,\lambda }+\sum_{\beta =E,I}\left(1-pf^{\beta }\right)C^{\alpha \beta }{\left(J_{-}^{\alpha \beta }\right)}^{2}\nu^{\beta ,p+1}\\ +C_{\text{ext}}^{\alpha E}{\left(J_{\text{ext}}^{\alpha E}\right)}^{2}\nu_{o}^{\alpha },\;\text{if}\;\gamma =[1,\ldots ,p]\\ \sum_{\beta =E,I}C^{\alpha \beta }f^{\beta }{\left(J_{-}^{\alpha \beta }\right)}^{2}\sum_{\lambda =1}^{p}\nu^{\beta ,\lambda }+\sum_{\beta =E,I}\left(1-pf^{\beta }\right)C^{\alpha \beta }{\left(J^{\alpha \beta }\right)}^{2}\nu^{\beta ,p+1}+C_{\text{ext}}^{\alpha E}{\left(J_{\text{ext}}^{\alpha E}\right)}^{2}\nu_{o}^{\alpha },\;\text{if}\;\gamma =p+1 \end{array}\right.$$

where $\nu^{\beta ,\lambda }$ is the firing rate of population (β,λ) with β∈{E,I}, λ∈{1,…,p+1}; all other parameters in Eqs. 14–15 are defined in Sec. IV B. For each population, μ and σ contain recurrent contributions from the same population and from the other populations in the network, as well as an external contribution from the background input. The system given by Eqs. 12, along with the threshold and reset conditions for the membrane potential, can be analyzed using the Fokker-Planck framework [94]. When $\tau_{s}<<\tau_{m}^{\alpha }$, the steady-state firing rate of neurons in population (α,γ) satisfies the self-consistent relationship

(16) $$\nu^{\alpha ,\gamma }={\text{Φ}}^{\alpha ,\gamma }\left[\mu^{\alpha ,\gamma }(\nu ),\sigma^{\alpha ,\gamma }(\nu )\right].$$

In Eq. 16, $\nu =\left[\nu^{E,1},\ldots ,\nu^{E,p+1},\nu^{I,1},\ldots ,\nu^{I,p+1}\right]$ is the vector of firing rates of each population and ${\text{Φ}}^{\alpha ,\gamma }$ is the transfer function for population (α,γ), given by

(17) $${\text{Φ}}^{\alpha ,\gamma }={\left[\tau_{r}+\tau_{m}^{\alpha }\sqrt{\pi }\int_{q_{r}^{\alpha ,\gamma }}^{q_{t}^{\alpha ,\gamma }}e^{x^{2}}\mathrm{erfc}(-x)dx\right]}^{-1}$$

where

(18) $$q_{r}^{\alpha ,\gamma }=\frac{V_{r}^{\alpha }-\tau_{m}^{\alpha }\mu^{\alpha ,\gamma }}{\sqrt{\tau_{m}^{\alpha }}\sigma^{\alpha ,\gamma }}+a\sqrt{\tau_{s}/\tau_{m}^{\alpha }}$$

(19) $$q_{t}^{\alpha ,\gamma }=\frac{V_{t}^{\alpha }-\tau_{m}^{\alpha }\mu^{\alpha ,\gamma }}{\sqrt{\tau_{m}^{\alpha }}\sigma^{\alpha ,\gamma }}+a\sqrt{\tau_{s}/\tau_{m}^{\alpha }}$$

and with $a=-\zeta (1/2)/\sqrt{2}$ [96].

To find allowed states of the network, we numerically solved the set of 2(p+1) self-consistent equations defined by Eq. 16 in conjunction with Eq. 14 and 15. Importantly, multiple solutions – corresponding to different numbers of active and inactive clusters – can exist for the same set of parameters. In such cases, the solution obtained will depend on the initial guess for firing rate vector. To systematically deal with this fact, we looked for solutions with $n_{A}$ active clusters and $p-n_{A}$ inactive clusters by setting the initial rates for the first $n_{A}$ E and first $n_{A}$ I populations to $\nu_{\text{high}}^{E}$ and $\nu_{\text{high}}^{I}$, respectively, and the initial rates for the remaining E and I populations to $\nu_{\text{low}}^{E}$ and $\nu_{\text{low}}^{I}$, respectively. By choosing $\nu_{\text{high}}^{E}>\nu_{\text{low}}^{E}$ and $\nu_{\text{high}}^{I}>\nu_{\text{low}}^{I}$ we biased the numerical solver to search for solutions with $n_{A}$ active clusters; the solution space was then be mapped by varying $n_{A}\in \{0,\ldots ,p\}$.

We denote a self-consistent solution with $n_{A}$ active clusters as $\nu_{n_{A}}$. The solution in which all clusters have the same firing rate (i.e., $n_{A}=0$) is referred to as the “uniform state” and solutions with $n_{A}\geq 1$ active clusters are referred to as “cluster states”. In the cluster states, the $n_{A}$ active clusters of type α∈{E,I} have steady-state rate $\nu_{n_{A},↑}^{\alpha }$ and the $p-n_{A}$ inactive clusters of type α have rate $\nu_{n_{A},↓}^{\alpha }$, where $\nu_{n_{A},↑}^{\alpha }>\nu_{n_{A},↓}^{\alpha }$. Depending on the network parameters, cluster states for a particular $n_{A}$ may or may not exist.

#### 2. In the presence of quenched variability

When ${\text{Δ}}_{H}^{E}\neq 0$, the mean background input to excitatory neurons varies across the population due to the quenched randomness in the external inputs (Sec. IV B 4). To perform a mean-field analysis under these conditions, the formalism can be adapted to account for the distribution of firing rates induced within each population as a result of the quenched variability [97–99]. The analysis proceeds by assuming that the spatial distribution of mean inputs to cells in population (α,γ) is Gaussian, with population average ${\bar{\mu }}^{\alpha ,\gamma }$ and population standard deviation ${\text{Δ}}^{\alpha ,\gamma }$ for α∈{E,I}, γ∈{1,…,p+1}. The population average ${\bar{\mu }}^{\alpha ,\gamma }$ is given by

(20) $${\bar{\mu }}^{\alpha ,\gamma }=\left\{\begin{array}{l} \sum_{\beta =E,I}C^{\alpha \beta }f^{\beta }J_{+}^{\alpha \beta }{\bar{\nu }}^{\beta ,\gamma }+\sum_{\beta =E,I}C^{\alpha \beta }f^{\beta }J_{-}^{\alpha \beta }\sum_{\begin{array}{c} \lambda =1\\ \lambda \neq \gamma \end{array}}^{p}{\bar{\nu }}^{\beta ,\lambda }+\sum_{\beta =E,I}\left(1-pf^{\beta }\right)C^{\alpha \beta }J_{-}^{\alpha \beta }{\bar{\nu }}^{\beta ,p+1}\\ +C_{\text{ext}}^{\alpha E}J_{\text{ext}}^{\alpha E}\nu_{o}^{\alpha }\;\text{if}\;\gamma =[1,\ldots ,p]\\ \sum_{\beta =E,I}C^{\alpha \beta }f^{\beta }J_{-}^{\alpha \beta }\sum_{\lambda =1}^{p}{\bar{\nu }}^{\beta ,\lambda }+\sum_{\beta =E,I}\left(1-pf^{\beta }\right)C^{\alpha \beta }J^{\alpha \beta }{\bar{\nu }}^{\beta ,p+1}+C_{\text{ext}}^{\alpha E}J_{\text{ext}}^{\alpha E}\nu_{o}^{\alpha }\;\text{if}\;\gamma =p+1, \end{array}\right.$$

where ${\bar{\nu }}^{\beta ,\lambda }$ is the spatially-averaged rate across cells in population (β,λ) with β∈{E,I}, λ∈{1,…,p+1}. The mean input to the $i^{\text{th}}$ cell in population (α,γ) can then be written as

(21) $$\mu_{i}^{\alpha ,\gamma }={\bar{\mu }}^{\alpha ,\gamma }+{\text{Δ}}^{\alpha ,\gamma }z_{i}$$

where $z_{i}\sim N(0,1)$. The spatial variance ${\text{Δ}}^{\alpha ,\gamma }$ of the mean inputs across population (α,γ) has contributions from the quenched randomness in the external input and from the induced spatial variability of the firing rates within each recurrent population. Taking into account these two sources, we have

(22) $${\left({\text{Δ}}^{\alpha ,\gamma }\right)}^{2}=\left\{\begin{array}{l} \sum_{\beta =E,I}C^{\alpha \beta }f^{\beta }{\left(J_{+}^{\alpha \beta }\right)}^{2}{\left(s^{\beta ,\gamma }\right)}^{2}+\sum_{\beta =E,I}C^{\alpha \beta }f^{\beta }{\left(J_{-}^{\alpha \beta }\right)}^{2}\sum_{\begin{array}{c} \lambda =1\\ \lambda \neq \gamma \end{array}}^{p}{\left(s^{\beta ,\lambda }\right)}^{2}+\sum_{\beta =E,I}\left(1-pf^{\beta }\right)C^{\alpha \beta }{\left(J_{-}^{\alpha \beta }\right)}^{2}{\left(s^{\beta ,p+1}\right)}^{2}\\ +{\left(C_{\text{ext}}^{\alpha E}J_{\text{ext}}^{\alpha E}{\text{Δ}}_{H}^{\alpha }\nu_{o}^{\alpha }\right)}^{2}\;\text{if}\;\gamma =[1,\ldots ,p]\\ \sum_{\beta =E,I}C^{\alpha \beta }f^{\beta }{\left(J_{-}^{\alpha \beta }\right)}^{2}\sum_{\lambda =1}^{p}{\left(s^{\beta ,\lambda }\right)}^{2}+\sum_{\beta =E,I}\left(1-pf^{\beta }\right)C^{\alpha \beta }{\left(J^{\alpha \beta }\right)}^{2}{\left(s^{\beta ,p+1}\right)}^{2}+{\left(C_{\text{ext}}^{\alpha E}J_{\text{ext}}^{\alpha E}{\text{Δ}}_{H}^{\alpha }\nu_{o}^{\alpha }\right)}^{2}\;\text{if}\;\gamma =p+1 \end{array}\right.$$

where ${\left(s^{\beta ,\lambda }\right)}^{2}$ is the spatial variance of the firing rates in population (β,λ) and where the last term is the contribution from the external inputs. As is typical, spatial heterogeneity of the input variance $\sigma^{\alpha ,\gamma }$ is neglected [97, 98]. In this way, $\sigma^{\alpha ,\gamma }$ is the same for all neurons in population (α,γ) and given by

(23) $${\left(\sigma^{\alpha ,\gamma }\right)}^{2}=\left\{\begin{array}{l} \sum_{\beta =E,I}C^{\alpha \beta }f^{\beta }{\left(J_{+}^{\alpha \beta }\right)}^{2}{\bar{\nu }}^{\beta ,\gamma }+\sum_{\beta =E,I}C^{\alpha \beta }f^{\beta }{\left(J_{-}^{\alpha \beta }\right)}^{2}\sum_{\begin{array}{c} \lambda =1\\ \lambda \neq \gamma \end{array}}^{p}{\bar{\nu }}^{\beta ,\lambda }+\sum_{\beta =E,I}\left(1-pf^{\beta }\right)C^{\alpha \beta }{\left(J_{-}^{\alpha \beta }\right)}^{2}{\bar{\nu }}^{\beta ,p+1}\\ +C_{\text{ext}}^{\alpha E}{\left(J_{\text{ext}}^{\alpha E}\right)}^{2}\nu_{o}^{\alpha }\;\text{if}\;\gamma =[1,\ldots ,p]\\ \sum_{\beta =E,I}C^{\alpha \beta }f^{\beta }{\left(J_{-}^{\alpha \beta }\right)}^{2}\sum_{\lambda =1}^{p}{\bar{\nu }}^{\beta ,\lambda }+\sum_{\beta =E,I}\left(1-pf^{\beta }\right)C^{\alpha \beta }{\left(J^{\alpha \beta }\right)}^{2}{\bar{\nu }}^{\beta ,p+1}+C_{\text{ext}}^{\alpha E}{\left(J_{\text{ext}}^{\alpha E}\right)}^{2}\nu_{o}^{\alpha }\;\text{if}\;\gamma =p+1 \end{array}\right.$$

The mean firing rates are parameterized by the standard Gaussian random variable z, and are determined self-consistently via

(24) $$\nu^{\alpha ,\gamma }(z)={\text{Φ}}^{\alpha ,\gamma }\left[\mu^{\alpha ,\gamma }\left(z,\bar{\nu },s^{2}\right),\sigma^{\alpha ,\gamma }\left(\bar{\nu },s^{2}\right)\right],$$

where ${\text{Φ}}^{\alpha ,\gamma }$ is defined by Eqs. 17–19, and where $\bar{\nu }=\left[{\bar{\nu }}^{E,1},\ldots ,{\bar{\nu }}^{E,p+1},{\bar{\nu }}^{I,1},\ldots ,{\bar{\nu }}^{I,p+1}\right]$ is the vector of average firing rates and $s^{2}=\left[{\left(s^{E,1}\right)}^{2},\ldots ,{\left(s^{E,p+1}\right)}^{2},{\left(s^{I,1}\right)}^{2},\ldots ,{\left(s^{I,p+1}\right)}^{2}\right]$ is the vector of firing rate variances. Finally, the across-population mean ${\bar{\nu }}^{\alpha ,\gamma }$ and variance ${\left(s^{\alpha ,\gamma }\right)}^{2}$ of the firing rates in population (α,γ) are given by

(25) $${\bar{\nu }}^{\alpha ,\gamma }=\frac{1}{\sqrt{2\pi }}\int dze^{-z^{2}/2}\nu^{\alpha ,\gamma }(z)$$

and

(26) $${\left(s^{\alpha ,\gamma }\right)}^{2}=\frac{1}{\sqrt{2\pi }}\int dze^{-z^{2}/2}{\left[\nu^{\alpha ,\gamma }(z)\right]}^{2}-{\left({\bar{\nu }}^{\alpha ,\gamma }\right)}^{2},$$

which must be solved for self-consistently in conjunction with Eq. 24. Akin to the analysis in the absence of quenched variability (Sec. IV L 1), we searched for solutions with a certain number of active clusters by varying the initial rates for the numerical solver. We denote a self-consistent solution with $n_{A}$ active clusters as the pair of vectors $\left({\bar{\nu }}_{n_{A}},s_{n_{A}}^{2}\right)$. As before, the uniform state corresponds to the case $n_{A}=0$, and is characterized by all clusters having the same population average rate. In cluster states (which have $n_{A}\geq 1$ active clusters), the active and inactive cluster rates are denoted by ${\bar{\nu }}_{n_{A},↑}^{\alpha }$ and ${\bar{\nu }}_{n_{A},↓}^{\alpha }$, respectively, where ${\bar{\nu }}_{n_{A},↑}^{\alpha }>{\bar{\nu }}_{n_{A},↓}^{\alpha }$ and α∈{E,I}.

#### 3. Selecting the E-to-E intracluster connection strength for the mean-field analyses

To study the effect of the ${\text{Δ}}_{H}^{E}$ arousal modulation in the mean-field theory, we first examined the effect of the E-to-E intracluster coupling strength $\left(J_{+}^{EE}\right)$, which controls the dynamical regime of the network [34]. We considered the standard scenario ${\text{Δ}}_{H}^{E}=0$, and varied $J_{+}^{EE}\in [12,19.5]$ using steps of size $\text{Δ}J_{+}^{EE}=0.025$. At each $J_{+}^{EE}$, we searched for self-consistent solutions $\nu_{n_{A}}$ with $n_{A}=[0,\ldots ,5]$ active clusters. Whether or not a cluster solution exists for a particular $n_{A}\geq 1$ depends on the value of $J_{+}^{EE}$ (Fig. S11A).

To compare to the mean-field theory, we ran an additional set of network simulations in which $J_{+}^{EE}$ was varied in the range [12,19.5] in steps of size $\text{Δ}J_{+}^{EE}=0.075$. For these simulations, no arousal modulations or sensory stimuli were applied, and we ran 20 trials per each of 5 network realizations; all other parameters were as described in Table S1 and Sec. IV B 1. For each simulated trial at a given $J_{+}^{EE}$, we computed *(i)* the active cluster rate $\nu_{n_{A},↑}^{E}$ conditioned on a given number of active clusters $n_{A}$ (Sec. IV H 2), *(ii)* the probability $P\left(n_{A}\right)$ of finding $n_{A}$ active clusters (Sec. IV H 2), and *(iii)* the population average firing rate of all E neurons. Analyses were based on 3.3 seconds of simulated activity per trial, and all quantities were averaged across trials and network realizations. Results are shown in Fig. S11B; note that the active cluster rate $\nu_{n_{A},↑}^{E}$ is only plotted for values of $n_{A}$ satisfying $P\left(n_{A}\right)\geq 0.1$.

We observed that cluster states emerged at lower values of $J_{+}^{EE}$ in the simulations compared to the mean-field (Fig. S11A,B). This is potentially due to the finite-size of the simulated networks and the inexact incorporation of synaptic dynamics in the mean-field. Although the mean-field does not quantitatively capture the behavior of the simulations, it can still provide insight into the effects of ${\text{Δ}}_{H}^{E}$. In order to qualitatively compare the theory and simulations as a function of ${\text{Δ}}_{H}^{E}$, we considered a fixed intracluster coupling for the simulations $\left(J_{+,\text{sim}}^{EE}\right)$, and then ran mean-field calculations at a larger intracluster coupling $J_{+,\text{mft}}^{EE}$ that gave the best match to the simulations run at $J_{+,\text{sim}}^{EE}$ in the absence of the arousal modulation $\left({\text{Δ}}_{H}^{E}=0\right)$. Specifically, we fixed $J_{\text{+,sim}}^{\text{EE}}=15.75$ (default value used throughout the main text), and computed the active cluster rate $\nu_{n_{A}^{*},↑,\text{sim}}^{E}\left[J_{+}^{EE}=15.75\right]$ conditioned on the most likely number of active clusters $n_{A}^{*}=3$. In the mean-field, we then determined the value of $J_{+}^{EE}$ for which the active cluster rate $\nu_{n_{A}^{*},↑,\text{mft}}^{E}\left[J_{+}^{EE}\right]$ most closely matched the value $\nu_{n_{A}^{*},↑,\text{sim}}^{E}\left[J_{+}^{EE}=15.75\right]$ from the simulations. This procedure yielded a mean-field intracluster coupling of $J_{+,\text{mft}}^{EE}=16.725$ (Fig. S11A), which was then used for the mean-field calculations performed as a function of ${\text{Δ}}_{H}^{E}$ in the main text (Fig. 5A).

#### 4. Mean-field analysis of clustered network dynamics as a function of the input heterogeneity

The mean-field analysis provides the steady-state firing rates of active and inactive clusters, conditioned on a particular number $n_{A}$ of active clusters. Together, these rates summarize the collective activity patterns of the network. To elucidate how the ${\text{Δ}}_{H}^{E}$ arousal modulation affects the dynamics of the clustered networks, we fixed the E-to-E intracluster coupling $J_{+}^{EE}$ according to the procedure in Sec. IV L 3; all other network parameters were set to the values given in Table. S1. For a particular choice of $n_{A}$, we then solved for the mean-field rates ${\bar{\nu }}_{n_{A},↑}$ and ${\bar{\nu }}_{n_{A},↓}$ (Sec. IV L 2) as a function of ${\text{Δ}}_{H}^{E}$; this process was then repeated for different numbers of active clusters $n_{A}$. In general, whether or not a cluster state solution is found for a particular $n_{A}\geq 1$ depended on ${\text{Δ}}_{H}^{E}$; for some values of ${\text{Δ}}_{H}^{E}$, only the uniform state was found (Fig. S12A).

In the main text, we examined excitatory mean-field rates ${\bar{\nu }}_{n_{A},↑}^{E}$ and ${\bar{\nu }}_{n_{A},↓}^{E}$ as function of the ${\text{Δ}}_{H}^{E}$ arousal modulation. (Sec. II D). In Fig. 5A, the rates are shown for the case of $n_{A}=3$ active clusters, which was the most frequently observed state in the simulations (Sec. IV H 2; Fig. S12C). If the cluster state solution was not found at a given value of ${\text{Δ}}_{H}^{E}$, then the rate corresponding to the uniform solution was plotted. In a supplementary analysis, we also show the rates for different values of $n_{A}$ separately as a function of ${\text{Δ}}_{H}^{E}$ (Fig. S12). Note that because the mean-field analysis used a different intracluster coupling than the simulations ($J_{+,\text{mft}}^{EE}\neq J_{+,\text{sim}}^{EE}$; Sec IVL4), the comparison between the mean-field and simulations in Fig. 5 is only meant to be qualitative.

### M. Effective mean-field theory of reduced 2-cluster networks

The mean-field theory presented in the previous section yields the steady-state cluster firing rates, but it cannot make predictions about dynamical transitions between the metastable states. To further understand the switching behavior of the clustered networks (Fig. 5D,E), we adapted the effective mean-field theory developed by [56] and utilized in [36, 48]. For these calculations, we analyzed a reduced version of the full LIF clustered networks composed of two excitatory clusters $E_{1}$ and $E_{2}$, one background (unclustered) excitatory population $E_{b}$, and one background inhibitory population $I_{b}$; this 2-cluster network was constructed as described in Sec. IV B 2, with the exception that we did not depress inter-cluster weights (see Table S2 for reduced network parameters). With the chosen parameters, the standard mean-field theory in the absence of the ${\text{Δ}}_{H}^{E}$ arousal modulation (Sec. IV L 1) predicts the presence of a uniform fixed point and two configurations in which one cluster is active and the other inactive (Fig. S13B). The effective MFT enables insight into dynamical transitions between the two cluster states via a dimensionality reduction process that results in a description of the cluster states as wells in an effective potential energy landscape.

Following Mascaro and Amit [56], the analysis proceeds by splitting the network’s populations into two groups: *(1)* a set of “in-focus” populations whose dynamical behaviors are of interest, and *(2)* a set of “ambient” populations. Here, the two clusters $E_{1}$ and $E_{2}$ are taken as the in-focus populations, and their rates $\nu^{F}$ are treated as parameters; $E_{b}$ and $I_{b}$ are taken as the ambient populations. For some frozen combination of the in-focus rates $\nu_{\text{in}}^{F}=\left(\nu_{\text{in}}^{E,1},\nu_{\text{in}}^{E,2}\right)$, the rates of the ambient populations $\nu^{A}=\left(\nu^{E,b},\nu^{I,b}\right)$ are allowed to adapt, and are computed self-consistently (Sec. IV L 1) by solving the coupled system of equations

(27) $$\nu^{E,b}={\text{Φ}}^{E,b}\left[\mu^{E,b}\left(\nu_{\text{in}}^{F},\nu^{A}\right),\sigma^{E,b}\left(\nu_{\text{in}}^{F},\nu^{A}\right)\right]$$

(28) $$\nu^{I,b}={\text{Φ}}^{I,b}\left[\mu^{I,b}\left(\nu_{\text{in}}^{F},\nu^{A}\right),\sigma^{I,b}\left(\nu_{\text{in}}^{F},\nu^{A}\right)\right].$$

Feedback from the ambient populations then induces new output rates $\nu_{\text{out}}^{F}=\left(\nu_{\text{out}}^{E,1},\nu_{\text{out}}^{E,2}\right)$ for the in-focus populations, which are given by

(29) $$\nu_{\text{out}}^{E,1}={\text{Φ}}^{E,1}\left[\mu^{E,1}\left(\nu_{\text{in}}^{F},\nu^{A}\right),\sigma^{E,1}\left(\nu_{\text{in}}^{F},\nu^{A}\right)\right]={\text{Φ}}_{\text{eff}}^{E,1}\left[\nu_{\text{in}}^{F}\right]$$

(30) $$\nu_{\text{out}}^{E,2}={\text{Φ}}^{E,2}\left[\mu^{E,2}\left(\nu_{\text{in}}^{F},\nu^{A}\right),\sigma^{E,2}\left(\nu_{\text{in}}^{F},\nu^{A}\right)\right]={\text{Φ}}_{\text{eff}}^{E,2}\left[\nu_{\text{in}}^{F}\right]$$

In Eqs. 27–30, the μ’s, σ’s, and Φ’s are computed similarly to Eqs. 14, 15, and 17, but adjusted for the 2-cluster system.

The induced rates $\nu_{\text{out}}^{F}$ are in general different from the initial rates $\nu_{\text{in}}^{F}$. By varying $\nu_{\text{in}}^{F}$ and computing the difference $\nu_{\text{out}}^{F}-\nu_{\text{in}}^{F}$ at each point, we obtain a flow map in the $\left(\nu_{\text{in}}^{E,1},\nu_{\text{in}}^{E,2}\right)$ plane (see Fig. S13C). This flow map captures the response of the clusters to a particular set of quenched input rates $\left(\nu_{\text{in}}^{E,1},\nu_{\text{in}}^{E,2}\right)$, and contains the effect of feedback from the ambient populations. In this way, the map reveals the system’s fixed points and the flow of the cluster rates $\nu^{E,1}$ and $\nu^{E,2}$ away from the stationary points. Examination of this reduced 2D description indicates that the two cluster states are attractors of the system, and are linked by an unstable fixed point corresponding to the uniform state ($\nu^{E,1}=\nu^{E,2}$; Fig. S13D).

To perform the effective MFT in the presence of the arousal modulation ${\text{Δ}}_{H}^{E}$, we neglected the influence of the firing rate variance $s^{2}$ (i.e., we set $s^{2}=0$ for all populations in Eq. 22; Sec. IV L 2) [36, 48]. With this simplification, Eq. 24 becomes

(31) $$\nu^{\alpha ,\gamma }(z)={\text{Φ}}^{\alpha ,\gamma }\left[\mu^{\alpha ,\gamma }(z,\bar{\nu }),\sigma^{\alpha ,\gamma }(\bar{\nu })\right],$$

where the $\mu^{\alpha ,\gamma }$, $\sigma^{\alpha ,\gamma }$, and ${\text{Φ}}^{\alpha ,\gamma }$ are computed similarly to Eqs. 21, 23, and 17, but adjusted for the 2-cluster system. Each population in the network is then described only by its population average rate ${\bar{\nu }}^{\alpha ,\gamma }$ (Eq. 25); note that for the 2-cluster network, α∈{E,I} and γ∈{1,2,b}. From this point, the 2D flow map in the ${\bar{\nu }}^{E,1}-{\bar{\nu }}^{E,2}$ plane can be computed using the dimensionality reduction procedure described above. We found that neglecting the spatial variance of the rates had only a small effect on the self-consistent solution for the population average rates $\bar{\nu }$.

To understand how the arousal modulation impacts the cluster dynamics, we performed the effective MFT for several values of ${\text{Δ}}_{H}^{E}$ (see Table S2). In each case, we obtained a compact representation of the system by numerically integrating the 2D flow-field along a trajectory connecting the two cluster states via the unstable fixed point (see Fig. S13C). This process results in a 1D effective potential with two wells – corresponding to the two cluster states – separated by a barrier whose maxima corresponds to the uniform state (Figs. 5D). The height h of this barrier controls the rate of stochastic transitions between the two cluster states [33, 36, 48, 57]. Computing the barrier height as a function of ${\text{Δ}}_{H}^{E}$ thus provides insight into the effects of ${\text{Δ}}_{H}^{E}$ on the cluster dynamics, with lower barriers indicating faster switching and shorter-lived cluster activation periods (Fig. 5E).

### N. Statistical analysis

Boxplots display the median and the first and third quartiles of the data, with the whiskers extending from the quartiles to ± 1.5 of the interquartile range. All statistical tests were non-parametric (Wilcoxon signed-rank test for paired data and Mann-Whitney U test for unpaired data).

## Supplementary Material

Supplement 1
